# Did the poor gain from India’s health policy interventions? Evidence from benefit-incidence analysis, 2004–2018

**DOI:** 10.1186/s12939-021-01489-0

**Published:** 2021-07-10

**Authors:** Sakthivel Selvaraj, Anup K. Karan, Wenhui Mao, Habib Hasan, Ipchita Bharali, Preeti Kumar, Osondu Ogbuoji, Chetana Chaudhuri

**Affiliations:** 1 Public Health Foundation of India, Gurugram, India; 2 Indian Institute of Public Health, Gurugram, India; 3 Centre for Policy Impact in Global Health, Duke University, Durham, NC 27708 USA

**Keywords:** Inequity, Utilization of health care, Public subsidy, Benefit-incidence analysis

## Abstract

**Background:**

Health policy interventions were expected to improve access to health care delivery, provide financial risk protection, besides reducing inequities that underlie geographic and socio-economic variation in population access to health care. This article examines whether health policy interventions and accelerated health investments in India during 2004–2018 could close the gap in inequity in health care utilization and access to public subsidy by different population groups. Did the poor and socio-economically vulnerable population gain from such government initiatives, compared to the rich and affluent sections of society? And whether the intended objective of improving equity between different regions of the country been achieved during the policy initiatives? This article attempts to assess and provide robust evidence in the Indian context.

**Methods:**

Employing Benefit-Incidence Analysis (BIA) framework, this paper advances earlier evidence by highlighting estimates of health care utilization, concentration and government subsidy by broader provider categories (public versus private) and across service levels (outpatient, inpatient, maternal, pre-and post-natal services). We used 2 waves of household surveys conducted by the National Sample Survey Organisation (NSSO) on health and morbidity. The period of analysis was chosen to represent policy interventions spanning 2004 (pre-policy) and 2018 (post-policy era). We present this evidence across three categories of Indian states, namely, high-focus states, high-focus north eastern states and non-focus states. Such categorization facilitates quantification of reform impact of policy level interventions across the three groups.

**Results:**

Utilisation of healthcare services, except outpatient care visits, accelerated significantly in 2018 from 2004. The difference in utilisation rates between poor and rich (between poorest 20% and richest 20%) had significantly declined during the same period. As far as concentration of healthcare is concerned, the Concentrate Index (CI) underlying inpatient care in public sector fell from 0.07 in 2004 to 0.05 in 2018, implying less pro-rich distribution. The CI in relation to pre-natal, institutional delivery and postnatal services in government facilities were pro-poor both in 2004 and 2018 in all 3 groups of states. The distribution of public subsidy underscoring curative services (inpatient and outpatient) remained pro-rich in 2004 but turned less pro-rich in 2018, measured by CIs which declined sharply across all groups of states for both outpatient (from 0.21 in 2004 to 0.16 in 2018) and inpatient (from 0.24 in 2004 to 0.14 in 2018) respectively. The CI for subsidy on prenatal services declined from approximately 0.01 in 2004 to 0.12 in 2018. In respect to post-natal care, similar results were observed, implying the subsidy on prenatal and post-natal services was overwhelmingly received by poor. The CI underscoring subsidy for institutional delivery although remained positive both in 2018 and 2004, but slightly increased from 0.17 in 2004 to 0.28 in 2018.

**Conclusions:**

Improvement in infrastructure and service provisioning through NHM route in the public facilities appears to have relatively benefited the poor. Yet they received a relatively smaller health subsidy than the rich when utilising inpatient and outpatient health services. Inequality continues to persist across all healthcare services in private health sector. Although the NHM remained committed to broader expansion of health care services, a singular focus on maternal and child health conditions especially in backward regions of the country has yielded desired results.

**Supplementary Information:**

The online version contains supplementary material available at 10.1186/s12939-021-01489-0.

## Introduction

Achieving the goal of equity – defined as substantive equality of opportunity - is considered an important economic justification for public policy intervention. It has been argued that the distribution of public subsidy and benefits of health services across population groups should be according to need for health care, and not on the basis of their ability to pay for care or place of residence or other social determinants [1, 2]. However, inequality in access to healthcare, utilization and financial protection remains one of the major concerns of the Low - and Middle-Income Counties (LMICs). WHO’s Commission on Social Determinants of Health (CSDH) identified large observable differences in health outcomes within and between countries that are determined by avoidable inequalities in the access to resources [3]. Theoretical framework proposed by Wagstaff suggests that increases in income inequality at individual level may result in higher levels of health inequality at the population level - countries with unequal income distributions are likely to have unequal distributions of health across income quintiles [4]. Furthermore, Contoyannis and Forster (1999a) have argued that health inequality will still rise when everyone’s income goes up by the same proportion, if the income elasticity of demand for health rises as income rises [5]. Theoretical framework points out that public provisioning of subsidized or free health care will reduce inequalities by increasing the likelihood of both the poor and the rich individual to improve their health, but the former is expected to gain a higher proportion due to diminishing returns in health production resulting in larger benefit accruing to them [4]. Sen’s capability approach to health equity emphasizes the multidimensional nature including the role played by fairness in delivery of health care among other determinants [6]. A model based analysis of 14 countries demonstrated that if investment levels were to be similar, an equity-driven intervention could substantially reduce child death and stunting besides improving cost-effectiveness of those interventions [7].

Besides a whole lot of theoretical literature, empirical evidence on health equity abounds. A multi-country study highlighted accelerating health inequalities despite rising per capita income, resulting from economic growth accompanied by technological change, as elites were able to assimilate new technology faster than economically poor ones [4]. Income inequality at the household level is often found to drive disparities in health outcomes, access and utilization. A recent Indian study revealed an absolute gap of 7.6 years in life expectancy at birth between the top richest and bottom poorest quintile groups [8]. Marked differences were found in skilled birth attendance in India during 2007–08, wherein merely 23.6% of poorest quintile could receive such care against 84.9% for the richest quintile [9]. Another study pointed to concentration of untreated morbidity among the vulnerable population, namely among poor and elderly population [10]. Several findings in the Indian context presenting distributional impact of subsidy using Benefit Incidence Analysis (BIA) highlighted imbalance in targeting and distribution of health subsidies across population groups and type of services. It was pointed out that the poor groups living in the economically backward states and rural areas received inadequate health subsidy as compared to rich counterparts. Moreover, it was also observed that the magnitude of subsidies and utilization patterns in hospital care were driving the maldistribution across population groups, especially for curative care service while better targeting for maternal and child health care services [11]. Noting skewed distribution of government spending on health across states, a study utilising 2004 and 1995–96 household data suggested mixed results with some states showing better targeting resulting in pro-poor allocation, while in large number of states the distribution remained pro-rich [12]. Employing similar data sets and the period of analysis involving two Indian states, evidence from Tamil Nadu and Odisha demonstrated a divergent result. With public subsidy turning pro-poor in 2004 compared to 1995–96 in the state of Tamil Nadu whereas in Odisha, inpatient episodes and maternity services remained regressive while outpatient services remained progressive [13]. A recent study using 2014 national survey data demonstrated that utilisation of child deliveries and inpatient services are relatively pro-poor as against outpatient visits that remained pro-rich [14]. .However, it also pointed out that when net and gross benefits are taken together, health care services tended to be relatively equal but less pro-poor.

India introduced a range of health sector reforms during the last decade and a half. Among others, these reforms included two key elements. As a supply side intervention, a national flagship programme called the National Rural Health Mission (NRHM) was initiated in the year 2005, aimed at strengthening the public healthcare delivery system, with a focus on improving maternal and child healthcare [15]. On the demand side, a fully subsidized health insurance scheme, *Rashtriya Swasthya Bima Yojana* (RSBY), initiated in 2007, primarily aimed at providing financial risk protection from hospitalization for India’s 300 million poor [16]. The NRHM – which was later revamped as National Health Mission (NHM) in 2013- was implemented in a ‘mission’ mode to achieve scale and speed of execution. The bottom-up planning approach under NHM replaced vertically-driven, top-down disease-specific health intervention to create a continuum of care with appropriate integration across level of cares in the public facilities. Though NHM focussed its efforts on all states, special attention was directed at High-Focus States. NRHM classified different Indian states under three groups: i) High-Focus States (HF); ii) High-Focus North-Eastern states (HFNE) and; iii) Non High-Focus States^1^ (Others). Since improving equity in maternal and child healthcare has been one of the important thrust under the scheme, states with higher fertility and high infant and maternal mortality rates were grouped as high focussed states [17]. Both financial and non-financial resources were allocated in a manner that relatively benefited the high-focus states. For instance, currently the contribution of funds to the scheme varies, where federal and state governments contribute in the ratio of 75:25% in high-focus states, whereas the respective share in the Non High-Focus States are in the proportion of 60:40% of fund allocation. One of the key programs under the NRHM was the *Janani Suraksha Yojana* (JSY), a conditional cash transfer programme to pregnant women which was created to encourage women to deliver in institutions rather than homes, to reduce maternal mortality.

Another, key government intervention was a demand side intervention through government-sponsored health insurance, RSBY, directed at poor and other less advantaged population which was expected to accelerate access to hospitalisation and provide financial risk protection. RSBY was later revamped as *Pradhan Mantri Jan Arogya Yojana* (PM-JAY) and integrated in the *Ayushman Bharat* Scheme initiated in 2018 [18]. The PMJAY sought to cover about 500 million poor and socio-economically vulnerable population. Before PMJAY was launched, the demand side intervention through publicly funded health insurance scheme, besides the federal scheme (RSBY), several state governments launched state specific insurance schemes either as complementary or alternative to the national level health insurance scheme. The state specific schemes provided wider population coverage and liberal benefit packages in contrast to RSBY’s targeted approach to population coverage and a limited benefit package. Evaluations of these schemes in general have shown that although schemes were not successful in providing financial risk protection, it significantly improved utilisation of healthcare among poor [19, 20].

India’s health spending (public and private) is estimated at 3.8% of its GDP during 2016–17 (National Health Systems Resource Centre (NHSRC) 2018). Government funding, which currently constitutes about one-third of all health expenditure, is funded by both central and state governments, with the latter accounting for nearly two-thirds of contribution. Prepaid and risk-pooled funds have been historically inadequate in India but has been on the rise from roughly 28% in 2004–05 to about 44% during 2016–17. Social insurance was limited to covering about 133 million. Donor funding, which used to be over 2% of all health spending during the early 2000, witnessed sharp drop which accounts for 0.6% in 2016–17. Although significant share of government funding has been directed at strengthening public sector facilities, over the last decade, there has been an effort to promote tax-funded health insurance programs targeted at poor and low-paid informal sector workers for providing free and cashless inpatient care services. Since the launch of NHM and government sponsored health insurance schemes, tax funded resources increased as a share of total health expenditure from approximately 23% during early 2000s to 32% in 2016–17. Admittedly, greater flexibility and predictability in central government’s funding has been achieved through off-treasury route, with frontline facilities receiving untied funds for routine use. Despite these measures, health expenditure is overwhelmingly financed by out-of-pocket (OOP) expenditure (around 59% of all health spending). Sustained underfunding of public sector facilities, and the rapid growth of private sector providers has contributed to a higher burden of out-of-pocket costs of health care for households. Of this, almost two-thirds of OOP expenses were directed at outpatient care, especially medicines. Over 55 million people are estimated to be impoverished annually on account of health care spending (Selvaraj et al. 2018).

This study aims to assess whether health financing reforms (through NHM and government funded health insurance) and accelerated public health investments in India was able to close the gap in inequality in healthcare utilization and access to public subsidy. The period of analysis was chosen to represent the reform period involving 2004 (pre-reform) and 2018 (post-reform era). We present this evidence across three categories of Indian states, namely, high-focus states, high-focus north eastern states and non-focus states. Such categorization facilitates quantification of reform impact of policy level interventions in three groups. Employing BIA framework, this paper advances earlier evidence by highlighting estimates of health utilization, concentration and subsidy by broader provider categories (public versus private) and across service levels (outpatient, inpatient, maternal, pre and post-natal services).

## Methods and data

### Data collection

We used 2 waves of household surveys conducted by the National Sample Survey Organisation (NSSO) on health and morbidity. These surveys were conducted by the NSSO during January–June 2004 and July 2017-June, 2018. The number of samples covered under the survey include 73,868 households (383,338 persons) in 2004 and 113,823 households (555,115 persons) in 2017–18 using a multistage stratified sampling process (See Table 1). Total samples were representative at the national and state level. The information was collected from selected households using a questionnaire schedule (25.0). In addition to a range of socio-economic identifiers, it collected detailed information on type of morbidities (self-reported), health care utilization and expenditure pattern across different types of healthcare facilities (government, private and non-government providers).

**Table 1:** Summary Statistics

Sample Indicators	2004	2018
Total Sample Population	3,83,338	5,55,115
Total Sample Households	73,868	1,13,823
Average Household Size	5.18	4.35
Median age (Years)	24	27
Educational Attainment		
No formal Schooling	34.13%	26.06%
Up to Primary Schooling	61.11%	29.96%
Middle/Secondary Schooling	3.81%	25.99%
Higher Secondary & above	0.95%	17.99%
Monthly Per Capita Consumption Expenditure (Rupees Nominal Terms)	₹1838	₹2617
Outpatient Visits in Govt. Facilities	22%	30%
Percent Hospitalisation in Govt. Hospitals	40%	42%
Percentage Child Birth in Govt. Hospitals	21%	47%
Percentage ANC (15–49 years)	73%	97%
Percentage PNC (15–49 years)	45%	88%

Source: National Sample Survey Organisation, Health and Morbidity Survey, 2004 and 2017–18

The NSSO schedule recorded response of individuals/households to specific questions eliciting information on healthcare utilization and reason for the same. The healthcare utilisation pattern recorded detailed information on number of days of ailments and hospitalisation during a recall period of 15 days and 1 year respectively. It further recorded detailed expenditure incurred by households on each episode of treatment of ailment besides maternity and child healthcare disaggregated by items of expenditure such as consultancy fee, hospital charges, drugs, diagnostics and use of medical appliances. The survey also provides information on non-medical expenditure such as transport, food and lodging expenses by households incurred for healthcare utilisation. Expenditure on all types of healthcare and maternity care were comparable across the two surveys. Households’ total consumption expenditure during a reference period of 1 month is recorded in the survey as ‘usual monthly consumption expenditure’. We used this information to generate quintile groups (5 equal division) of population weighted by number of episodes of healthcare/maternity care. Although the survey captures a detailed facility level data in each public and private category, we have reported only government and private facility as a single group in each category. The parameters and characteristics described above is often considered to be a gold-standard approach in successfully conducting BIA studies [21].

### Analytical approaches

We employed standard methods of Benefit Incidence Analysis (BIA) which estimates share of healthcare utilisation and public subsidy distributed across different income groups of population and the related index of inequality, defined as ‘concentration index’ [22, 23].

### Concentration index

The concentration index (CI) is an index of the distribution of an outcome which is defined as:

1 $$ CI=\frac{2}{\mu}\ast \mathit{\operatorname{cov}}\left(h,r\right) $$

Where ‘μ’ is mean outcome and ‘cov(h,r)’ is covariance between the healthcare/subsidy outcome and the fractional rank in the living standards distribution. The value of CI is restricted to the range (− 1, 1). A positive (negative) value indicates that the rich (poor) have better healthcare/subsidy distribution than the poor (rich) have. If no socio-economic inequality in utilisation of healthcare/subsidy is observed, the concentration index will turn to zero [23].

We estimated separate concentration indices for each outcome indicator for the years 2004 and 2017–18 to reflect unadjusted changing scenario of inequality over the period under consideration. We also plotted concentration curves to depict changing inequality in different outcome indicators over the same period. Concentration curve lying above (below) the line of equality represent pro-poor (rich) distribution of healthcare/subsidy.

### Outcome indicators

We generated a range of outcome indicators representing healthcare utilisation: i) outpatient visits, ii) hospital admissions, iii) prenatal care, iv) postnatal care and v) institutional child delivery. These outcome indicators were analysed separately for public and private facilities. In addition, we also estimated the amount of public subsidy utilised by population underlying these 5 outcome indicators.

### Estimation of public subsidy

In the next step, the magnitude of public subsidy was estimated for each type of healthcare separately for the year 2004 and 2017–18, reflecting difference between per episode OOP payments at public and private facilities. Such differences in OOP payments between public and private facilities were generated at disaggregated level by allowing range of variations represented by geographical region (6 regions comprised of groups of states), rural-urban areas of residence of population (2 groups), quintile groups of population (5 groups), ailment types (19 groups including prenatal, postnatal and child delivery), days of ailment (hospitalisation) of ailing persons (3 groups) and age groups of ailing persons. Altogether we allowed more than 5000 levels of variations (representing different groups of population) for estimating the difference in OOP payments between public and private facilities. Using these parameters, we predicted (using limited depended variable regression model [logit model]) the private facility equivalent OOP payments to represent public facility healthcare utilisation (See Supplementary Table S1.a and S1b. for regression results).

Finally, the difference between the actual OOP payments and the predicted values for the public facilities represented the public subsidy per episode of healthcare/maternity care. As a robustness check, we cross-checked the predicted values for private facilities and the difference between actual OOP payments at the private facility and the predicted values that were in a range of 0.2 to 2.3% for different quintile groups (Supplementary Table S2). This method generated distribution of public subsidy across population utilising public healthcare services as outpatient, inpatient, prenatal, postnatal and child delivery. This distribution was used to estimate proportion of public subsidy shared by each quintile groups while concentration indices were estimated using the Eq. (1) for each of the healthcare utilisation indicator.

## Results

The key results presented in this section highlights changes in equity dimension underlying health care utilisation, concentration of utilisation and concentration of public subsidy among income classes. Results presented here pertain to the periods 2004 and 2017–18, across quintile groups of population involving groups of states categorised as i) High Focus (HF), ii) High Focus North East (HFNE) and iii) Other (OTH) states.

### Health care utilisation

**Fig. 1:**
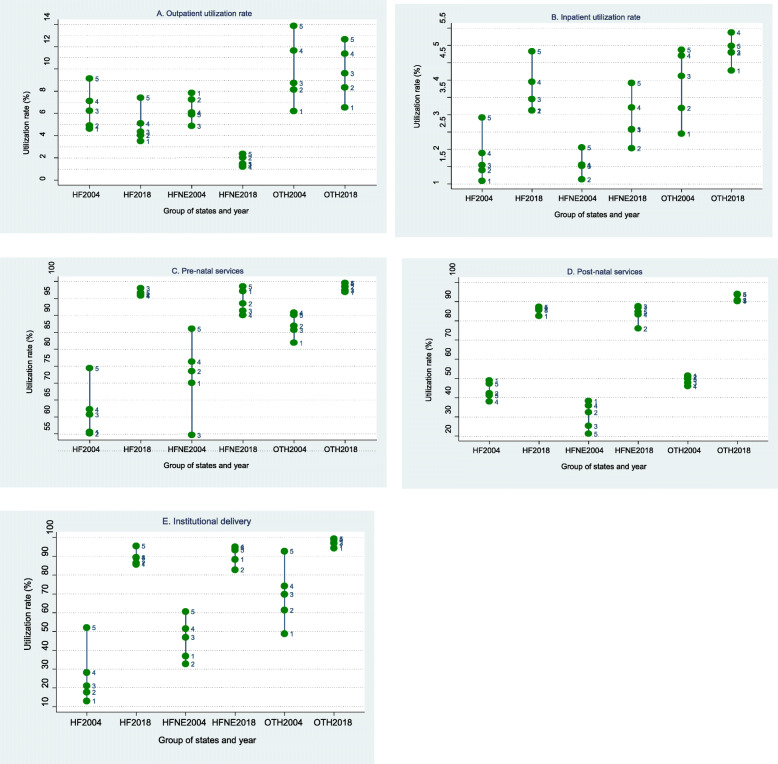
(**A**-**E**): Healthcare utilisation rate (%) in high focus, high focus north east and other states by quintile groups of population, 2004 and 2017–18. Notes: 1. Numbers 1 to 5 on the vertical lines represent quintile groups of population with 1 being the lowest and 5 being the highest 20%; 2. HF is high focus, HFNE is high focus north east, OTH is other states and suffixes 2004 and 2018 are the reference years

In respect to health care utilisation rates (proportion of population using healthcare), we outline a range of healthcare services (outpatient, inpatient, prenatal care, postnatal care and institutional delivery (Fig. 1) and its distribution by quintile groups of population along with concentration indices. In the interests of clarity of reporting, we present results only for the lowest and highest 20% of population by living standard status, separately for the states categorised in the 3 groups and all the states taken together (All India). Detailed results for all the quintile groups of population are presented in the annexure.

The evidence revealed that health care utilisation, except outpatient care, accelerated significantly in 2018 as compared to 2004. Also, the gap in the utilisation rates between the poor and rich (particularly between the poorest 20% and the richest 20%) has significantly declined during the same period. Detailed results with mean utilisation rates among all quintile groups, along with 95% confidence intervals are outlined in Supplementary Tables S3 and S4.

### Inpatient and outpatient care

In general, utilization of outpatient care declined in 2018 as compared to 2004 across the groups of states and quintile groups of population, except among the lowest 3 quintile groups in the other states category (Fig. 1A). However, the decline was sharper among the richer quintile groups leading to narrowing gap between utilization of outpatient care across the quintile groups in 2018. For instance, decline in the utilization of outpatient care among the richest quintile was 1.6 percentage points (3.5%age points in the high focus north east states) as against 0.7 among the poorest 20%. The highest declines were witnessed in the HFNE states across all the quintile groups.

In respect to hospital based inpatient care, although all quintile classes in 3 groups of states recorded an increase in utilisation rates, the difference between quintile groups remains quite stark. For instance, in 2018, hospitalisation rate was about 3.1% (increased from 1.1% in 2004) among poorest quintile population as against 4.8% (increased from 2.9% in 2004) among the richest quintile classes (Fig. 1B). Similarly, the increase in inpatient utilization rate was 1 percentage points as against approximately 2 percentage points increase recorded among the richest quintile groups.

### Maternity care

As far as the maternity healthcare is concerned, all three basic maternity care services, prenatal, institutional delivery and postnatal reflected not only significant jump in utilization rates but also reduction in gaps between rich and poor in using maternal services during 2004–18 (Fig. 1C, D and E) (see also Supplementary Table S4).

### Health care concentration

#### Inpatient care

Health care concentration of inpatient episodes and outpatient visits are highlighted in Table 2. It may be observed that the concentration of public inpatient healthcare (% share of all inpatient care) among the lowest 20% of population declined slightly from 19% in 2004 to 18% in 2018. While the same remained constant among richest 20% by during the period under consideration. Accordingly, the Concentration Index (CI) is estimated to have declined from 0.07 in 2004 to 0.05 in 2018, signifying increased share of inpatient care in favour of poor. In the high-focus states, the concentration of public inpatient care accelerated by more than 6.5 percentage points (13.8% in 2004 to 20.4% in 2018) among the poorest 20% while the same declined by 3 percentage points among the richest 20%. The relative CI declined from 0.14 in 2004 to 0.09 in 2018. However, the trend is mixed among the HFNE states. The CI involving public inpatient care in the HFNE states was closer to zero (0.019) in 2004 which increased to 0.17 in 2018 although the poorest 20% continued to contribute marginally higher share of public inpatient care as compared to richest 20% population. In the non-focus states (OTH), the CI was negative (*− 0.031)* in 2004, signifying distribution of the public inpatient care in favour of poor, which further improved to − 0.08 in 2018.

**Table 2:** Percentage of inpatient and outpatient healthcare shared by the poorest 20% and the richest 20% population groups and concentration indices in 2004 and 2018

Quintile Groups	2004				2018			
	HF States	HFNE States	Other States	All states	HF States	HFNE States	Other States	All states
**Inpatient Public**								
Poorest 20%	13.77	20.32	20.15	19.03	20.37	19.7	23.41	18.07
Richest 20%	26.46	20.34	14.85	21.82	23.29	16.5	14.27	21.83
*CI*	0.141	0.019	−0.031	0.07	0.096	0.164	− 0.080	0.049
*SE*	0.011	0.029	0.010	0.00	0.012	0.013	0.009	0.007
**Inpatient Private**								
Poorest 20%	10.95	17.43	12.14	10.01	15.11	13.62	14.84	11.9
Richest 20%	35.56	38.95	28.71	37.51	35.52	43.91	28.1	33.14
*CI*	0.245	0.291	0.224	0.283	0.248	0.356	0.170	0.245
*SE*	0.011	0.048	0.007	0.006	0.011	0.024	0.007	0.006
**Outpatient Public**								
Poorest 20%	18.49	25.64	19.78	18.47	20.42	21.6	23.02	15.95
Richest 20%	28.8	16.3	18.01	26.71	24.59	12.62	17.26	28.32
*CI*	0.158	−0.109	0.020	0.102	0.091	−0.100	0.004	0.125
*SE*	0.016	0.038	0.012	0.01	0.024	0.061	0.013	0.011
**Outpatient Private**								
Poorest 20%	13.17	23.83	12.63	14.09	15.32	17.65	13.27	11.64
Richest 20%	27.2	20.9	29.12	31.31	29.11	34.32	31.46	31.9
*CI*	0.143	−0.045	0.196	0.191	0.163	0.161	0.179	0.229
*SE*	0.008	0.029	0.006	0.005	0.015	0.077	0.009	0.008

Presumably, inpatient care in private facilities, remains highly pro-rich as the CIs in all three groups of states and national level are highly positive, although the non-focus states (OTH) showed a decline in the concentration index from 0.22 in 2004 to 0.17 in 2018. Also, the HFNE states signified increased concentration of inpatient care involving private facilities among the richest 20%, CI rising from 0.29 in 2004 to 0.35 in 2018 (The Share of all five quintiles in each groups of states are highlighted in Supplementary Table S5).

#### Outpatient care

Utilisation of outpatient care in government facilities reflected a mixed picture. While the public outpatient care utilisation was observed to be pro-rich during 2004 (CI 0.16) in HF states, the same was pro-poor in HFNE states (CI − 0.11). The distribution marginally improved in favour of poor in 2018 with CI in HF declining to 0.09 and in HFNE to − 0.10. Overall (all India) public outpatient care utilisation remained equally distributed across the population groups during 2004–2018. Distribution of outpatient care utilisation in private sector, which was pro-rich in 2004 (except in HFNE states with CI being − 0.05, i.e. pro-poor) turned relatively more pro-rich 2018 with CIs in all the three groups of states and all India increasing in the positive direction, reflecting pro-rich distribution.

#### Maternity care

Next, we highlight distribution (concentration) of maternity services (pre-natal, institutional delivery and post-natal) disaggregated by public and private provisions in Table 3. Concentration of pre-natal services in government facilities were observed to be pro-poor both in 2004 and 2018 with signs of concentration indices being negative (except in HFNF states in 2004). Nonetheless, in HF states in 2004, the poorest 20% shared more than one-fourth of pre-natal care as against the richest 20% sharing less than one-sixth of care. Moreover, the distribution turned further pro-poor in 2018 as reflected by the higher negative values of CIs in all three categories of states and national level. In contrast to maternity care utilised in public care, prenatal care involving private facilities was highly concentrated among rich as reflected by high positive values of CIs in all the three groups of states and national level during both time periods. In addition, the positive values of CIs increased in 2018 as against 2004, signifying higher inequality in utilising maternity care in private sector, favouring the rich (The Shares of all quintiles across three groups of states are presented in Supplementary Table S6).

**Table 3:** Percentage of maternity care shared by the poorest 20% and the richest 20% population groups and concentration indices in 2004 and 2018

Quintile Groups	2004				2018			
	HF States	HFNE States	Other States	All states	HF States	HFNE States	Other States	All states
**Pre-natal care in Public Facilities**								
Poorest 20%	22.38	23.35	28.5	24.91	28.61	25.22	29.33	26.87
Richest 20%	14.72	21.17	8.93	16.34	13.5	9.97	7.75	12.47
*CI*	*− 0.022*	*0.001*	*−0.174*	− 0.090	*− 0.126*	*− 0.073*	*−0.241*	− 0.212
*SE*	*0.021*	*0.051*	*0.017*	0.012	*0.013*	*0.027*	*0.014*	0.008
**Pre-natal care in Private Facilities**								
Poorest 20%	16.85	18.86	13.34	17.11	17.14	8.53	16.79	14.31
Richest 20%	24.99	36.52	24.18	22.67	30.11	36.17	22.61	25.36
*CI*	*0.073*	*0.197*	*0.138*	*0.114*	*0.153*	*0.354*	*0.073*	*0.146*
*SE*	*0.025*	*0.103*	*0.021*	*0.017*	*0.024*	*0.053*	*0.016*	*0.013*
**Institutional delivery in Public Facilities**								
Poorest 20%	17.49	18.98	26.41	22.43	29.61	23.05	29.46	27.5
Richest 20%	21.61	14.69	7.63	12.98	12.36	10.27	6.64	12.26
*CI*	*0.104*	*0.042*	*−0.193*	*−0.034*	*−0.142*	*−0.050*	*−0.258*	*−0.221*
*SE*	*0.042*	*0.069*	*0.021*	*0.019*	*0.012*	*0.026*	*0.010*	*0.007*
**Institutional delivery in Private Facilities**								
Poorest 20%	11.97	17.39	13.61	14.72	17.3	12.9	16.42	15.29
Richest 20%	37.88	32.67	25.11	27.87	32.86	49.15	23.61	27.07
*CI*	*0.286*	*0.068*	*0.183*	*0.261*	*0.176*	*0.370*	*0.092*	*0.164*
*SE*	*0.037*	*0.185*	*0.024*	*0.020*	*0.024*	*0.061*	*0.014*	*0.012*
**Post-natal care in Public Facilities**								
Poorest 20%	22.04	28.83	29.65	26.31	26.82	25	29.48	27.24
Richest 20%	16.74	13.8	8.93	14.04	17	10.27	6.27	11.96
*CI*	*−0.035*	*−0.072*	*− 0.215*	*− 0.119*	*− 0.142*	*−0.063*	*− 0.250*	*−0.218*
*SE*	*0.031*	*0.074*	*0.022*	*0.017*	*0.012*	*0.027*	*0.012*	*0.008*
**Post-natal care in Private Facilities**								
Poorest 20%	26.46	30.12	18.84	24.91	29.21	13.24	16.6	15.79
Richest 20%	17.27	15.72	20.37	17.04	12.95	44.1	24.28	26.25
*CI*	*−0.069*	*−0.129*	*0.066*	*−0.037*	*0.129*	*0.300*	*0.086*	*0.135*
*SE*	*0.025*	*0.126*	*0.028*	*0.020*	*0.022*	*0.068*	*0.014*	*0.012*

CIs for institutional delivery in public sector in 2018 were estimated to be invariably negative, echoing pro-poor concentration. The same was only negative in non-focus states in 2004. Similarly, post-natal care in government facilities is not only pro-poor but its distribution has improved in favour of poor in 2018 as against 2004, particularly in HF and OTH states besides the national level. On the other hand, institutional delivery and post-natal care in private sector remained pro-rich in 2018 as it was in 2004, although the trend suggests some improvements in access to institutional delivery for poor in the private sector.

### Concentration of public subsidy

Further, we observed that the distribution of public subsidy was highly pro-rich in 2004 both for inpatient and outpatient care (except for outpatient care in HFNE states), with the poorest receiving 14% and the richest cornering 29% of total public subsidy allocated to inpatient care, with CI being 0.24 (Table 4). The inequality in the distribution of subsidy for inpatient in 2004 was more prominent in HF states (CI being 0.33). Yet, inpatient subsidy remained pro-rich in 2018, although CIs declined sharply in all groups of states implying an improved distribution in favour of poor in relation to the year 2004. In respect to outpatient care, the Concentration Index underlying distribution of public subsidy in 2004 varied in a range of 0.85 in HFNE states to 0.29 in HF states. Subsidy contribution in outpatient care too signified improved distribution in favour of poor in 2018. Overall, the CI of public subsidy underscoring outpatient care declined from 0.20 in 2004 to 0.18 in 2018, entailing improved distribution of public subsidy on outpatient care in favour of poor led by HF and non-focus (OTH) states during both the period under consideration (The Shares of all quintiles across three groups of states are presented in Supplementary Table S7).

**Table 4:** Percentage of healthcare subsidy by the poorest 20% and the richest 20% population groups and concentration indices in 2004 and 2018

Quintile Groups	2004				2018			
	HF States	HFNE States	Other States	All States	HF States	HFNE States	Other States	All states
**Inpatient care**								
Poorest 20%	10.95	12.10	13.35	14.59	12.78	13.25	21.06	13.10
Richest 20%	37.81	29.89	24.21	29.48	34.89	32.93	23.17	26.68
Total	100	100	100	100	100	100	100	100
CI	*0.329*	*0.210*	*0.117*	*0.247*	*0.202*	*0.181*	*0.150*	*0.202*
SE	*0.016*	*0.042*	*0.013*	*0.010*	*0.013*	*0.029*	*0.011*	*0.009*
**Outpatient care**								
Poorest 20%	12.47	16.34	17.93	15.22	16.13	25.43	19.21	16.95
Richest 20%	34.05	27.85	27.75	31.78	28.02	22.52	23.42	30.16
Total	100	100	100	100	100	100	100	100
CI	*0.297*	*0.085*	*0.102*	*0.205*	*0.055*	*0.178*	*0.067*	*0.181*
SE	*0.029*	*0.052*	*0.018*	*0.014*	*0.022*	*0.099*	*0.013*	*0.018*

**Fig. 2:**
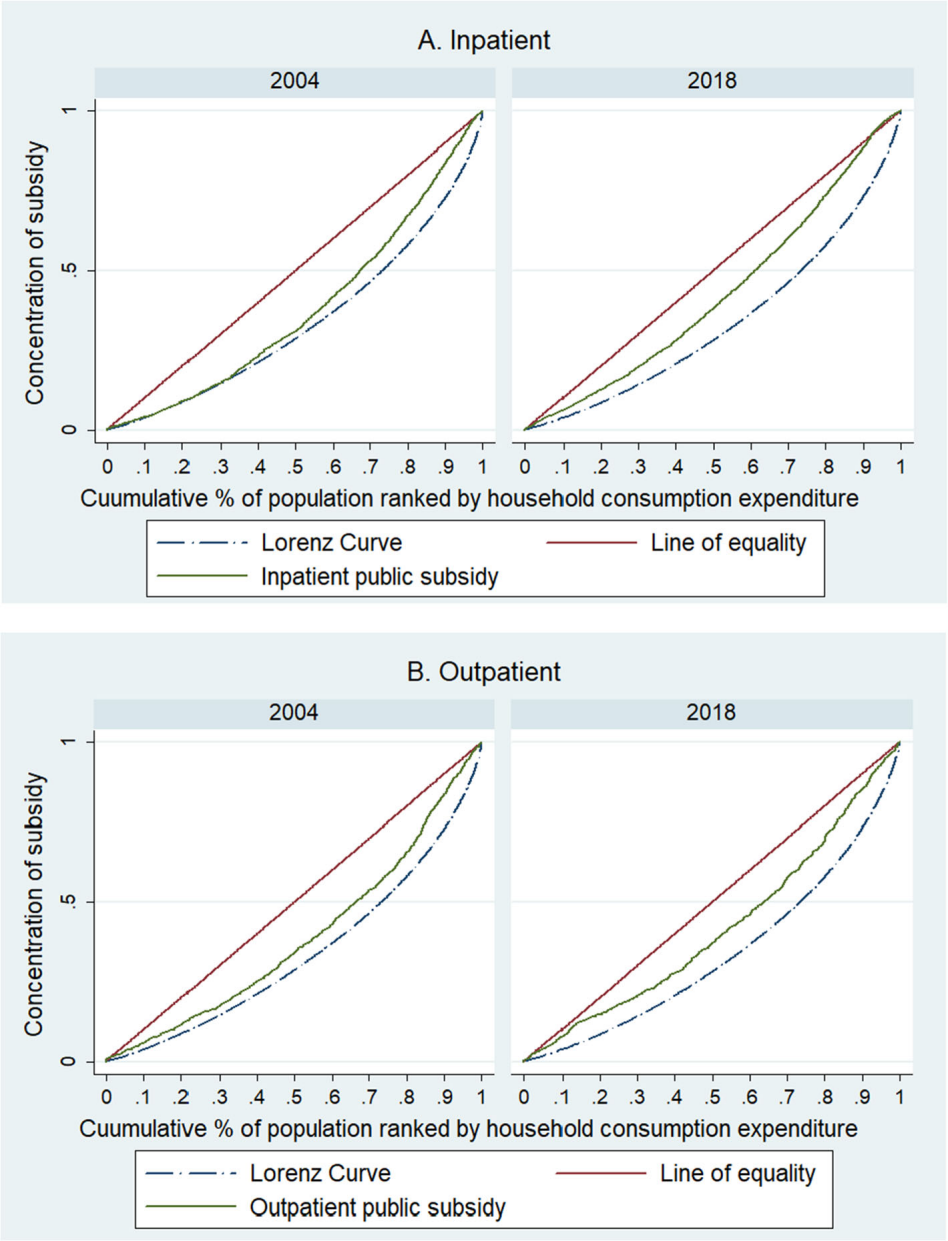
**A**-**B** Concentration of Public Subsidy on Inpatient and Outpatient Care in 2004 and 2018

The narrowing gap in distribution of public subsidy for inpatient and outpatient care over the period under consideration is also presented in Fig. 2. The concentration curves for inpatient care (Fig. 2A) and outpatient care (Fig. 2B) were closer to the line of equality in 2018 as against the year 2004.

Changing inequality in distribution of subsidy on maternal care (prenatal care, institutional delivery and postnatal care) is highlighted in Table 5. It may be observed that the indicators underlying CIs are positive both in 2018 and 2004, which was noted to be either positive. At the all India level, the CI for subsidy on prenatal services declined from approximately 0.01 in 2004 to 0.12 in 2018 implying that the subsidy on prenatal services was overwhelmingly received by poor. The largest change in the distribution of prenatal care subsidy was observed among HFNE states (CI declining from 0.43 in 2004 to 0.21 in 2018).

**Table 5:** Percentage of maternity care subsidy received by poorest 20% and richest 20% population and concentration indices in 2004 and 2018

Quintile Groups	2004				2018			
	HF States	HFNE States	Other States	All states	HF States	HFNE States	Other States	All states
Prenatal care								
Poorest 20%	16.20	1.90	20.73	19.05	26.39	11.39	25.80	20.68
Richest 20%	17.09	35.89	12.64	23.88	15.68	19.14	9.65	18.04
Total	100	100	100	100	100	100	100	100
CI	*0.085*	*0.436*	*0.070*	*0.014*	*0.118*	*0.213*	*0.066*	*0.128*
SE	*0.013*	*0.030*	*0.008*	*0.008*	*0.005*	*0.009*	*0.005*	*0.003*
Institutional delivery								
Poorest 20%	17.03	2.29	22.94	16.48	23.40	3.73	24.57	18.01
Richest 20%	17.97	16.33	12.23	24.68	17.84	19.76	13.04	19.69
Total	100	100	100	100	100	100	100	100
CI	*0.134*	*0.377*	*0.117*	*0.171*	*0.159*	*0.296*	*0.142*	*0.288*
SE	*0.010*	*0.027*	*0.009*	*0.007*	*0.008*	*0.011*	*0.008*	*0.005*
Postnatal care								
Poorest 20%	20.52	2.44	27.34	19.12	24.23	29.79	27.76	23.20
Richest 20%	17.54	20.40	8.68	22.72	15.45	22.73	7.27	15.19
Total	100	100	100	100	100	100	100	100
CI	*0.105*	*0.396*	*0.045*	*0.135*	*0.028*	*0.056*	*0.044*	*0.063*
SE	*0.028*	*0.037*	*0.013*	*0.014*	*0.008*	*0.018*	*0.006*	*0.005*

CIs for maternity care was estimated all within women of age between 15 and 49 years

Similarly, the CI involving subsidy on institutional delivery, although remained positive in 2018 signifying pro-rich distribution, the same declined from 0.15 in 2004 to 0.04 in 2018. Admittedly, the non-focus states recorded the highest decline in inequality (CI declining from 0.13 in 2004 to − 0.06 in 2018) in subsidy distribution on institutional delivery. However, HF and HFNE states too reported declining CIs during the same period. For postnatal care, the CIs of subsidy were negative in 2018 in all the three groups of states including national level, which were noted to be positive (except the non-focus states) in 2004. The largest change in inequality in favour of poor was in HF states (CI declining from 0.10 in 2004 to − 0.08 in 2018) followed by HFNE states (CI declining from 0.08 in 2004 to − 0.05 in 2018). (The respective shares of five quintiles across three groups of states are highlighted in Supplementary Table S8).

**Fig. 3:**
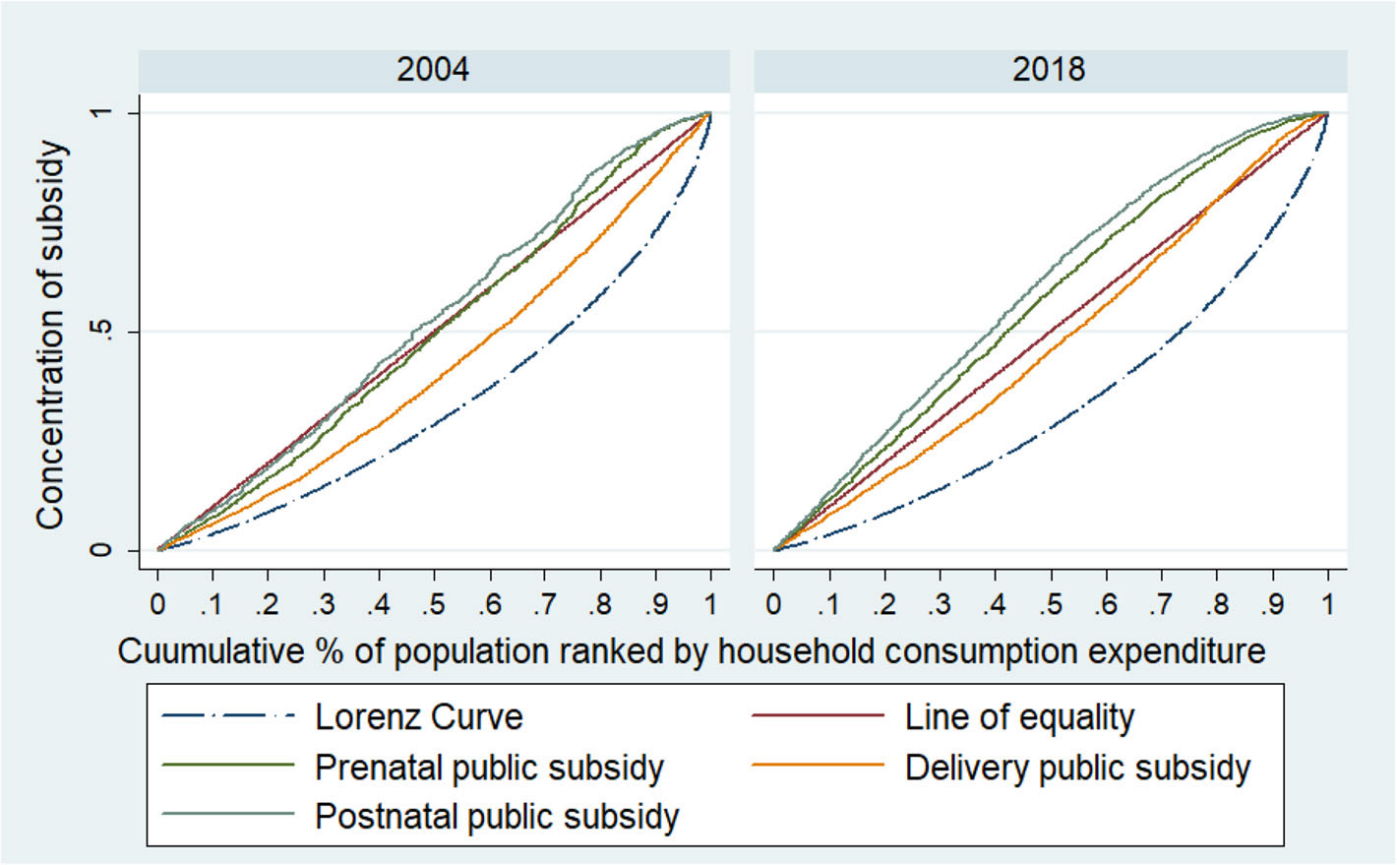
Concentration of Public Subsidy on prenatal care, institutional delivery and postnatal care in 2004 and 2018

Figure 3 highlights the changing inequality in concentration involving maternity care during the study period. The concentration curves of prenatal and postnatal cares which were close to the line of equality in 2004 shifted above the line of equality in 2018 mirroring the distributions to be pro-poor in contrast to the year 2004. The concentration curve of institutional delivery which was below the line of equality in 2004 moved closer to the line of equality in 2018 implying improved distribution of subsidy on institutional care favouring poor.

## Discussion

Our analysis demonstrates that utilisation of healthcare services, except outpatient care visits, accelerated significantly in 2018 from 2004. The difference in utilisation rates between poor and rich (between poorest 20% and richest 20%) had significantly declined during the same period. As far as concentration of healthcare is concerned, results suggest that the CI underlying inpatient care in public sector fell from 0.07 in 2004 to 0.05 in 2018, implying less pro-rich distribution. Whereas it remained pro-rich in private facilities, as the CIs in all the three groups of states and national averages were highly positive. On the other hand, outpatient care in public sector were equally distributed across the population groups during 2004–2018 while the distribution in private facilities, which remained pro-rich in 2004 (except in HFNE states) continued to be pro-rich in 2018 with positive CIs in all three groups of states. A related evidence from India for the period 2014 using similar data confirmed that public subsidy involving inpatient care has a pro-rich distribution. However, for the outpatient care, subsidy share was found to be higher among the richest in urban areas but highest among poorest class in the rural region [24]. Earlier, in another study Ghosh et al. had reported that for 2004, the magnitude of inequity among most Indian states involving outpatient and inpatient care was pro-rich across rural and urban areas. They also reported that horizontal inequity was much higher among rural as compared to urban population, for any type of curative care [25]. Using two periods of national sample survey data, our results demonstrate the changing trend of inequality in favour of poor involving inpatient care in public facilities. Evidence from sub-Saharan Africa and Asia-Pacific also suggests pro-rich distribution health care benefits in spite of progressive financing. Only few Asian countries - Thailand, Malaysia and Sri Lanka – achieved pro-poor distribution of health care benefits and progressive financing. A systematic review reported that the distribution of benefits at the primary health care level favored the poor while hospital level services benefited the better-off in majority of case [26].

Our analysis further demonstrates increased utilisation of maternal health care services (prenatal, institutional delivery and postnatal) during 2004–18. Concentration of pre-natal, institutional delivery and postnatal services in government facilities were pro-poor both in 2004 and 2018 in all 3 groups of states. However, the concentration of maternal healthcare in private sector remained highly concentrated among rich across three groups of states both in 2004 and 2018. Using 2014 sample survey data, Bower et al. reported similar results wherein inpatient and maternal health related delivery services were relatively pro-poor in public sector. The study reported that child delivery were pro-poor (concentration index − 0.200) whereas inpatient care utilization was slightly pro-poor (concentration index− 0.056) [14].

Emerging evidence in this paper underlying concentration index and public subsidy indicates that *Janani Suraksha Yojana (JSY)* – a conditional cash transfer scheme under NHM - may have contributed to reducing inequality involving maternal healthcare services (antenatal, delivery and postnatal). The core focus of the JSY was intended to improve institutional deliveries. It may be observed that JSY which was initiated during 2005 and in a span of 12 years, about nine in ten deliveries in rural India and 96% in urban areas occurred in a health facility [27] and majority of these deliveries were taking place in government health facilities [28]. Furthermore, previous research had confirmed the impact of referral transport services, an intervention under JSY, could have also facilitated improvement in institutional deliveries [29]. Other studies have confirmed that the share of institutional deliveries has accelerated far more rapidly in poorer Indian states than their higher-income counterparts resulting in greater decline in inter-state inequalities [30]. This analysis indicates that these distributional benefits are primarily driven by the HFNE states involving curative services and maternal health care. This is further reconfirmed from our results that shows distribution of the public subsidy on prenatal services (0.01 in 2004 to 0.12 in 2018) and postnatal services (0.13 in 2004 to 0.06 in 2018) turned pro-poor at national level. Yet, public subsidy on institutional delivery remained pro-rich even in 2018, with a slight rise in magnitude (0.17 in 2004 to 0.28 in 2018) was observed across states highlighting regional disparities [14]. Few recent evidence suggest that despite conditional cash transfer, nearly 98% pregnant women ended up paying for child delivery at private hospitals and about 56% in public health facilities [31].

Findings from this research further reveals that the distribution of public subsidy underscoring curative services (inpatient and outpatient) remained pro-rich in 2004 but turned less pro-rich in 2018, measured by CIs which declined across all groups of states for both outpatient (from 0.20 in 2004 to 0.18 in 2018) and inpatient (from 0.24 in 2004 to 0.20 in 2018) respectively. Evidence from a study from north eastern region of China using data from China’s National Health Services Survey (2003 and 2008, *N* = 27,239) reveals that government health services involving inpatient and outpatient were pro-rich, entailing continuing inequity in distribution of public subsidy [32]. A Bangladesh study demonstrated a pro-rich (CI = 0.237) distribution of healthcare benefits, largely driven by private providers, as there was little evidence of inequity in benefits from public (CI = 0.044) and non-governmental sector (CI = 0.095) providers [33]. One of the reasons for the persistent inequality in curative services (outpatient and inpatient), both in terms of utilisation and distribution of public subsidy in Indian states, could be limited reach of government-funded health insurance program because of imprecise and inappropriate targeting of beneficiaries [34] resulting in low enrollment of poorer population at household level [35] and district level [36], and accompanying poor service utilization and weak financial risk protection mechanisms [19]. A systematic review of health insurance studies for India reported improvement in enrolment but no clear trend of declining OOP expenditures [37].

Inequity in health care utilization and its distributional impact of public subsidy is often influenced by variation in health needs, differences in quantity of services used, poor quality of care resulting in weak demand and the dimensions of care avoidance. We performed additional analysis to account for variations in health needs by highlighting the differences in uptake of health care services and public subsidy by quintile groups among those seeking treatment for communicable and non-communicable disease conditions. It is often found that the poor tend to disproportionately get affected by communicable diseases whereas the rich face a relatively higher risk for non-communicable disease conditions. For instance, the need and therefore the utilisation of health care by poor involving communicable disease conditions are relatively larger both in public and private facilities while the rich had demanded far more care for non-communicable disease conditions. As a result, the healthcare subsidy that rich received is relatively far greater for non-communicable diseases while for the poor, the communicable disease conditions relatively predominated (Table S9 and S10 in the appendix highlights healthcare utilisation and public subsidy for communicable and non-communicable diseases). Similarly, it may be observed that the need (and demand) for maternity care is far greater among poorest than the richest population, as revealed by fertility rates that are more than double among the former than the latter [38]. While the health care need is far greater among poor, they frequently failed to get adequate treatment due to poor quality or non-availability of health care services. For instance, the national sample survey suggested that when the poor did not seek treatment nearly 18% was because a specified service was unavailable in an inpatient facility. Whereas, unsatisfactory quality was identified as a key reason by 40% of poor for not seeking treatment in an inpatient setting and over one-fourth in an outpatient setting (Supplementary Table S11 in the annexure).

### Limitations

Our findings should be interpreted with caution as we do not attribute pro-poor utilisation and public health subsidy as entirely driven by government policy and program (NHM) interventions alone. Health care utilisation and the benefits of public subsidy distribution among population groups can be influenced by several socio-economic and demographic factors. Since this research remained focussed on income and regional groupings, other vital factors remained unaddressed. For instance, women’s education plays a critical role in positively influencing maternal health care utilisation, as educated women have relatively better access to information [39]. Another Indian study alluded to education, caste and wealth to be the key predictors of utilization of health care services underlying the choice between government or private facilities [40]. Similarly, quality of care that women receive is also influenced by socio-economic conditions in which they live. Women who are poor, illiterate and belonging to rural areas were less likely to receive ante-natal care [41]. The BIA as an analytical tool does not capture quality of care, but a recent paper fills this gap by generating quality scores into the BIA [2]. Even though the national survey we used in this research gathered one set of question around quality of care in public health facilities, we have been able to highlight the quality dimension without associating any causal relationship. While utilisation and public subsidy remains the central theme of this paper, we did not assess inequality in healthcare need. Due to lack of a parameter in the survey data to measure health care need directly, we examined it through an indirect manner in this paper. Moreover, patients often end up paying informal/illegal user fees, especially at the government hospitals. Although this dimension assumes importance in estimating public subsidy, but in the absence of data in the survey, we were unable to account for the informal payments, thus undercounting the actual burden of public subsidy on the poor.

## Conclusion

It may be conclusively stated that the level of inequality in utilisation and concentration of healthcare between richest and poorest population has declined during 2004–2018 for underlying maternal health care as well as inpatient care in the government facilities. Improvement in infrastructure and service provisioning through NHM route in the public facilities appears to have relatively benefited the poor. However, these improvements in infrastructure and services do not adequately account for quality issues that continue to persist. And yet the poor received a relatively smaller health subsidy than the rich when utilising inpatient and outpatient health services. As far as private health care is concerned, inequality continues to persist across all healthcare services. Although the NHM remained committed to broader expansion of health care services, a singular focus on maternal and child health conditions especially in backward regions of the country has yielded desired results. However, to close the continuing gap in inequity in curative care services and provide financial risk protection to households, augmenting tax funded resources is the need of the hour. Both central and state governments may need to accelerate per capita spending on health, in order to achieve the target of public spending on health to reach 2.5 percent of GDP from its current level of over 1 %. A rise in spending must be accompanied by improving governance of frontline facilities to enhance their absorptive capacity and equitable distribution of resources across regions and population groups.

## Supplementary Information

**Additional file1: Table S1a.** Regression results of predictors of per episode inpatient and outpatient medical expenditure (INR) in private sector. **Table S1b.** Regression results of predictors of per episode child delivery, prenatal and postnatal care medical expenditure (INR) in private sector. **Table S2.** Per episode actual and estimated expenditure (INR) on healthcare/maternity care in private and public sector, 2004 and 2018 (at 2018 prices). **Table S3.** Percentage of population utilising healthcare as inpatients and outpatients across high focus, high focus north east and other states, 2004 and 2018. **Table S4.** Percentage of pregnant women utilising ante-natal care, post-natal care and institutional delivery across high focus, high focus north east and other states, 2004 and 2018.

**Additional file2: Table S5.** Percentage of inpatient and outpatient healthcare shared by the poorest 20% and the richest 20% population groups and concentration indices in 2004 and 2018. **Table S6.** Percentage of maternity healthcare shared by the poorest 20% and the richest 20% population groups and concentration indices in 2004 and 2018. **Table S7.** Percentage of healthcare subsidy by the poorest 20% and the richest 20% population groups and concentration indices in 2004 and 2018. **Table S8.** Percentage of maternity care subsidy received by poorest 20% and richest 20% population and concentration indices in 2004 and 2018. **Table S9.** Percentage of inpatient and outpatient healthcare shared by the poorest 20% and the richest 20% population groups and concentration indices in 2018. **Table S10.** Percentage of healthcare subsidy by the poorest 20% and the richest 20% population groups and concentration indices in 2018. **Table S11.** Distribution of Reasons for not availing healthcare treatment in Government Facilities Across Different Income Classes.

## Data Availability

Raw data is in public domain since the data is a government survey conducted during 2017–18. Supplementary tables and figures are enclosed as annexure.

## Footnotes

1 NRHM identified 18 focus states out of 34 Indian states for improving health status and strengthen the weak public heath institutions. This was sought to be achieved by focusing its attention on rural masses, especially poor women and children. The 18 High-Focus were further classified as 10 High-Focus large states (Bihar, Madhya Pradesh, Chhattisgarh, Odisha, Uttar Pradesh, Jammu & Kashmir, Rajasthan, Himachal Pradesh, Jharkhand and Uttarakhand) and 8 High-Focus North-Eastern States (Assam, Tripura, Meghalaya, Manipur, Nagaland, Mizoram and Sikkim).
