# Supplementary material for: Did the poor gain from India’s health policy interventions? Evidence from benefit-incidence analysis, 2004–2018
Source: Int J Equity Health. 2021 Jul 10;20:159. doi: 10.1186/s12939-021-01489-0 (PMC8272306; doi:10.1186/s12939-021-01489-0)
Supplement: Supplementary file 1 — Additional file1: Table S1a. Regression results of predictors of per episode inpatient and outpatient medical expenditure (INR) in private sector. Table S1b. Regression results of predictors of per episode child delivery, prenatal and postnatal care medical expenditure (INR) in private sector. Table S2. Per episode actual and estimated expenditure (INR) on healthcare/maternity care in private and public sector, 2004 and 2018 (at 2018 prices). Table S3. Percentage of population utilising healthcare as inpatients and outpatients across high focus, high focus north east and other states, 2004 and 2018. Table S4. Percentage of pregnant women utilising ante-natal care, post-natal care and institutional delivery across high focus, high focus north east and other states, 2004 and 2018. [file 12939_2021_1489_MOESM1_ESM.docx]

Supplementary Table S-I.a. Regression results of predictors of per episode inpatient and outpatient medical expenditure (INR) in private sector

| Dependent variable – per episode medical expenditure | Inpatient# | | Outpatient# | |
| --- | --- | --- | --- | --- |
|  | Coefficient | Std. Err. | Coefficient | Std. Err. |
| 1. Geographical region$ – Comparison group is North East states |  |  |  |  |
| Region North | 503 | 2998 | -315* | 171 |
| Region West | -5086* | 2914 | -291* | 168 |
| Region East | -4414 | 2933 | -298* | 168 |
| Region South | -8704*** | 2899 | -369* | 168 |
| Region Central | -7586** | 2914 | -169 | 168 |
| 1. Disease conditions – comparison group is other conditions |  |  |  |  |
| Fever | -20044*** | 2989 | -1381*** | 215 |
| Vector borne | -19218*** | 3208 | -1364*** | 234 |
| Cancers | 42241*** | 3459 | 1825*** | 332 |
| Blood disease | -14682*** | 3595 | -50 | 253 |
| Diabetes /Metabolic | -13263*** | 3373 | -1638*** | 215 |
| Mental /Neurological | -1984 | 3146 | -1054*** | 222 |
| Eye /Ear | -8952** | 3297 | -1319*** | 242 |
| Cardiovascular | 13170*** | 3066 | -1674*** | 214 |
| Respiratory | -13750*** | 3272 | -1546*** | 216 |
| Gastrointestinal | -9188*** | 3050 | -968*** | 220 |
| Musculo-Skeletal | 3694 | 3187 | -1465*** | 217 |
| Genito-Urinary | -8032** | 3107 | -142 | 241 |
| Obstetric | -9761*** | 3353 | 9 | 333 |
| Injuries | -1543 | 3035 | -533* | 244 |
| Others | -3758 | 3313 | -1240*** | 222 |
| 1. Quintile groups – comparison group is the richest 20% |  |  |  |  |
| Poorest 20% | -12759*** | 850 | -239*** | 46 |
| Second poorest 20% | -13025*** | 847 | -103* | 45 |
| Middle | -11648*** | 805 | -221*** | 42 |
| Second richest 20% | -9750*** | 715 | -128*** | 37 |
| 1. Rural | -7271*** | 578 | -86** | 31 |
| 1. Hospital stay days – comparison is >=10 days |  |  |  |  |
| <=3 days | -55272*** | 789 | NA | NA |
| 4-9 days | -40883*** | 755 | NA | NA |
| 1. Reference days of illness – comparison is >=14 days |  |  |  | 47 |
| <=4 days | NA | NA | -517*** | 48 |
| 5-14 days | NA | NA | -75 |  |
| _cons | 96620*** | 4131 | 2809*** | 270 |
| R-sq | 0.188 | | 0.045 | |
| # of observations | 42,858 | | 24,661 | |

Notes: * significant at 10% level; ** significant at 5% level; *** significant at 1% level; 2. standard errors in are mentioned in parentheses; 3. Standard errors clustered at district level; 4. All estimates are adjusted for covariates. # Excluding child birth cases. $ Geographical region: North- Chandigarh, Delhi, Haryana, Himachal Pradesh, Jammu & Kashmir, Punjab, Uttaranchal; West- Dadra & Nagar Haveli, Daman & Diu, GOA, Gujarat, Maharashtra, Rajasthan; East- Bihar, Chhattisgarh, Jharkhand, Odisha, West Bengal; South- Andhra Pradesh, Andaman & Nicobar Island, Lakshadweep, Karnataka, Kerala, Puducherry, Tamilnadu, Telangana; Mdhya Pradesh, Uttar Pradesh; NE states- Arunachal Pradesh, Assam, Manipur, Mizoram, Meghalaya, Nagaland, Sikkim, Tripura.

Source: Authors’ estimates using NSSO 2017018.

Supplementary Table S-Ib.: Regression results of predictors of per episode child delivery, prenatal and postnatal care medical expenditure (INR) in private sector

|  | Delivery | | Pre | | Post | |
| --- | --- | --- | --- | --- | --- | --- |
| exp_medical | Coefficient | Std. Err. | Coefficient | Std. Err. | Coefficient | Std. Err. |
| 1. Geographical region$ – Comparison group is North East states |  |  |  |  |  |  |
| Region North | 2522 | 2750 | 2088*** | 720 | -3500*** | 557 |
| Region West | -1775 | 2666 | 1528** | 683 | -3984*** | 537 |
| Region East | -4189 | 2680 | -594 | 684 | -4141*** | 538 |
| Region South | -3180 | 2652 | 3061*** | 680 | -3023*** | 535 |
| Region Central | -2597 | 2663 | 445 | 687 | -3044*** | 536 |
| 1. Type of delivery |  |  |  |  |  |  |
| Caesarean | 6489*** | 807 |  |  |  |  |
| Other | 20412*** | 3122 |  |  |  |  |
| 1. Quintile groups – comparison group is the richest 20% |  |  |  |  |  |  |
| Poorest 20% | -9749*** | 870 | -2129*** | 250 | -913*** | 177 |
| Second poorest 20% | -9530*** | 864 | -1716*** | 253 | -541*** | 177 |
| Middle | -9612*** | 813 | -1726*** | 234 | -539*** | 170 |
| Second richest 20% | -7853*** | 729 | -196 | 216 | -318** | 153 |
| 1. Rural | -5282*** | 589 | -493*** | 174 | -26 | 123 |
| 1. Hospital stay days – comparison is >=10 days |  |  |  |  |  |  |
| <=3 days | -44408*** | 924 |  |  |  |  |
| 4-9 days | -31952*** | 886 |  |  |  |  |
| 1. Age groups (years) – comparison is >=45 |  |  |  |  |  |  |
| 15-29 | -4562*** | 839 | -3689** | 1518 | 703 | 1021 |
| 30-44 | -2541*** | 788 | -3564** | 1524 | 711 | 1025 |
| _cons | 69994*** | 2910 | 10302*** | 1656 | 6181*** | 1144 |
| R-sq | 0.20 |  | 0.05 |  | 0.02 |  |
| # of observations | 79,746 |  | 9.973 |  | 8,756 |  |

Notes: * significant at 10% level; ** significant at 5% level; *** significant at 1% level; 2. standard errors in are mentioned in parentheses; 3. Standard errors clustered at district level; 4. All estimates are adjusted for covariates. # Excluding child birth cases. $ Geographical region: North- Chandigarh, Delhi, Haryana, Himachal Pradesh, Jammu & Kashmir, Punjab, Uttaranchal; West- Dadra & Nagar Haveli, Daman & Diu, GOA, Gujarat, Maharashtra, Rajasthan; East- Bihar, Chhattisgarh, Jharkhand, Odisha, West Bengal; South- Andhra Pradesh, Andaman & Nicobar Island, Lakshadweep, Karnataka, Kerala, Puducherry, Tamilnadu, Telangana; Mdhya Pradesh, Uttar Pradesh; NE states- Arunachal Pradesh, Assam, Manipur, Mizoram, Meghalaya, Nagaland, Sikkim, Tripura.

Source: Authors’ estimates using NSSO 2017-18.

Supplementary Table S-II: Per episode actual and estimated expenditure (INR) on healthcare/maternity care in private and public sector, 2004 and 2018 (at 2018 prices^[[1]](#footnote-1)^)

|  | 2004 | | | | 2018 | | | |
| --- | --- | --- | --- | --- | --- | --- | --- | --- |
|  | Private | | Public | | Private | | Public | |
|  | Actual | Estimated | Actual | Estimated | Actual | Estimated | Actual | Estimated |
| Inpatient |  |  |  |  |  |  |  |  |
| poorest | 13690 | 13809 | 6268 | 15721 | 23,439 | 23,919 | 2,913 | 14,597 |
| 2nd poorest | 15802 | 15731 | 6429 | 18615 | 23,261 | 23,803 | 3,159 | 16,011 |
| middle | 18431 | 18383 | 8789 | 22505 | 24,899 | 24,939 | 3,473 | 17,519 |
| 2nd richest | 21994 | 22047 | 9879 | 23635 | 27,980 | 28,577 | 3,899 | 21,628 |
| richest | 35021 | 35002 | 15478 | 38173 | 40,110 | 40,517 | 6,222 | 36,554 |
| Total | 23353 | 23356 | 9331 | 23677 | 29,738 | 30,147 | 3,754 | 19,996 |
| Outpatient |  |  |  |  |  |  |  |  |
| poorest | 556 | 566 | 142 | 574 | 643 | 667 | 366 | 643 |
| 2nd poorest | 632 | 643 | 903 | 640 | 781 | 809 | 541 | 786 |
| middle | 645 | 651 | 108 | 637 | 669 | 671 | 395 | 678 |
| 2nd richest | 830 | 830 | 308 | 798 | 765 | 788 | 437 | 785 |
| richest | 1075 | 1075 | 780 | 974 | 900 | 889 | 554 | 921 |
| Total | 801 | 801 | 479 | 714 | 774 | 784 | 456 | 759 |
| Pre-natal care |  |  |  |  |  |  |  |  |
| poorest | 1709 | 1641 | 511 | 1933 | 5,383 | 5,447 | 2,987 | 5,570 |
| 2nd poorest | 2858 | 2800 | 469 | 2910 | 6,127 | 6,169 | 3,934 | 6,532 |
| middle | 2692 | 2665 | 701 | 2607 | 5,786 | 5,821 | 3,159 | 6,202 |
| 2nd richest | 2934 | 2913 | 1053 | 2755 | 7,394 | 7,510 | 5,473 | 7,537 |
| richest | 3919 | 3898 | 1867 | 3637 | 7,651 | 7,740 | 4,373 | 7,567 |
| Total | 2947 | 2900 | 824 | 2657 | 6,616 | 6,690 | 4,040 | 6,756 |
| Institutional delivery |  |  |  |  |  |  |  |  |
| poorest | 7206 | 7151 | 1952 | 7040 | 17,601 | 18,069 | 1,517 | 9,636 |
| 2nd poorest | 9223 | 9194 | 2494 | 8694 | 18,530 | 18,910 | 1,762 | 10,696 |
| middle | 10445 | 10451 | 3313 | 9905 | 18,798 | 19,104 | 1,703 | 10,016 |
| 2nd richest | 13869 | 13893 | 3782 | 13053 | 24,258 | 24,637 | 1,832 | 13,381 |
| richest | 17080 | 17069 | 4483 | 16495 | 27,216 | 27,500 | 2,403 | 18,281 |
| Total | 12666 | 12658 | 3063 | 10419 | 22,186 | 22,538 | 1,760 | 11,539 |
| Post-natal care |  |  |  |  |  |  |  |  |
| poorest | 1138 | 1148 | 432 | 1283 | 2,471 | 2,497 | 2,056 | 2,358 |
| 2nd poorest | 1388 | 1385 | 521 | 1501 | 2,793 | 2,851 | 1,087 | 2,675 |
| middle | 1738 | 1741 | 1003 | 1780 | 2,771 | 2,786 | 2,133 | 2,765 |
| 2nd richest | 1741 | 1725 | 990 | 1738 | 3,025 | 3,121 | 2,098 | 2,894 |
| richest | 2162 | 2157 | 1543 | 2070 | 3,433 | 3,436 | 1,937 | 3,339 |
| Total | 1612 | 1609 | 809 | 1614 | 2,960 | 2,997 | 1,887 | 2,830 |

Supplementary Table S-III: Percentage of population utilising healthcare as inpatients and outpatients across high focus, high focus north east and other states, 2004 and 2018

|  | 2004 |  |  |  | 2017-18 |  |  |  |
| --- | --- | --- | --- | --- | --- | --- | --- | --- |
|  | High Focus | North East | Other | All India | High Focus | North East | Other | All India |
| **Outpatient** |  |  |  |  |  |  |  |  |
| Poorest 20% | 5.0 [4.8;5.2] | 9.0 [8.1;99] | 6.5 [6.2;6.8] | 5.6 [5.5;5.8] | 3.8 [3.7;4.0] | 2.2 [1.9;2.4] | 6.7 [6.4;7.0] | 4.4 [4.3;4.6] |
| 2nd poorest | 5.2 [5.0;5.5] | 6.7 [6.0;7.3] | 7.4 [7.1;7.8] | 6.2 [6.1;6.4] | 4.4 [4.2;4.6] | 1.5 [1.3;1.7] | 8.1 [7.9;8.4] | 5.8 [5.6;5.9] |
| middle | 6.7 [6.4;7.0] | 7.1 [6.6;7.6] | 8.5 [8.2;8.8] | 7.6 [7.4;7.8] | 5.2 [4.9;5.4] | 1.4 [1.2;1.6] | 8.4 [8.2;8.7] | 6.6 [6.5;6.8] |
| 2nd richest | 7.4 [7.1;7.7] | 4.9 [4.5;5.2] | 9.6 [9.3;9.9] | 8.5 [8.3;8.7] | 5.1 [4.9;5.4] | 1.3 [1.1;1.5] | 9.2 [8.9;9.4] | 7.4 [7.3;7.6] |
| Richest 20% | 9.8 [9.4;10.1] | 6.4 [5.9;6.8] | 14.1 [13.7;14.4] | 12.5 [12.3;12.7] | 7.7 [7.4;80] | 2.3 [2.1;2.5] | 12.2 [12.0;12.4] | 10.8 [10.6;11,0] |
| Total | 6.4 [6.3;6.5] | 6.4 [6.2;6.6] | 9.7 [9.6;9.8] | 8.1 [8.0;8.2] | 4.8 [4.7;50] | 1.7 [1.6;1.8] | 9.5 [9.4;9.6] | 7.0 [6.9;7.1] |
| **Inpatient** |  |  |  |  |  |  |  |  |
| Poorest 20% | 1.3 [1.2;1.4] | 1.8 [1.4;2.2] | 2.6 [2.4;2.8] | 1.8 [1.7;1.9] | 3.2 [3.1;3.6] | 2.8 [2.5;3.1]] | 4.8 [4.5;5.0] | 3.6 [3.5;3.7] |
| 2nd poorest | 1.5 [1.4;1.6] | 1.5 [1.2;1.8] | 3.2 [3.0;3.4] | 2.2 [2.1;2.3] | 3.3 [3.2;3.6] | 2.2 [1.9;2.4]] | 4.3 [4.2;4.5] | 3.7 [3.6;3.8] |
| middle | 1.7 [1.6;1.9] | 1.2 [1.0;1.4] | 3.5 [3.3;3.7] | 2.6 [2.5;2.7] | 3.7 [3.5;3.9] | 2.5 [2.3;2.8] | 4.8 [4.6;4.9] | 4.2 [4.1;4.3] |
| 2ndrichest | 2.0 [1.8;2.1] | 1.5 [1.3;1.7] | 4.0 [3.8;4.1] | 3.1 [2.9;3.2] | 4.1 [3.9;4.3] | 3.1 [2.9;3.4] | 4.7 [4.5;4.9] | 4.4 [4.3;4.6] |
| Richest 20% | 3.1 [2.9;3.3] | 1.8 [1.6;2.1] | 5.3 [5.1;5.5] | 4.5 [4.4;4.6] | 5.2 [5.0;5.4] | 4.2 [3.9;4.5] | 5.2 [5.1;5.4] | 5.2 [5.1;5.3] |
| All | 1.8 [1.7;1.8] | 1.6 [1.4;1.7] | 3.9 [3.8;4.0] | 2.8 [2.8;2.9 | 3.7 [3.6;3.7] | 2.9 [2.7;3.0 | 4.8 [4.7;4.9] | 4.2 [4.1;4.2] |

Notes: Figures in parentheses are 95% confidence intervals

Supplementary Table S-IV: Percentage of pregnant women utilising ante-natal care, post-natal care and institutional delivery across high focus, high focus north east and other states, 2004 and 2018

|  | 2004 |  |  |  | 2017-18 |  |  |  |
| --- | --- | --- | --- | --- | --- | --- | --- | --- |
|  | High Focus | North East | Other | All India | High Focus | North East | Other | All India |
| Pre-natal care |  |  |  |  |  |  |  |  |
| poorest | 55.6 | 70.0 | 81.9 | 67.7 | 95.8 | 97.1 | 96.9 | 96.3 |
| 2nd poorest | 55.1 | 73.5 | 86.9 | 69.6 | 96.1 | 93.5 | 98.5 | 97.1 |
| middle | 60.7 | 54.7 | 85.7 | 71.8 | 98.0 | 91.4 | 97.4 | 97.5 |
| 2ndrichest | 62.3 | 76.3 | 90.8 | 74.6 | 95.9 | 90.1 | 99.5 | 96.9 |
| richest | 74.4 | 86.0 | 90.1 | 81.5 | 96.7 | 98.6 | 99.5 | 97.9 |
| All | 61.0 | 72.5 | 86.9 | 72.6 | 96.4 | 94.2 | 98.1 | 97.0 |
| Post-natal care |  |  |  |  |  |  |  |  |
| poorest | 48.9 | 38.2 | 51.5 | 49.7 | 82.5 | 86.8 | 90.1 | 85.6 |
| 2nd poorest | 42.2 | 32.4 | 50.7 | 45.8 | 86.3 | 76.0 | 90.7 | 87.9 |
| middle | 41.1 | 25.3 | 47.7 | 43.6 | 85.6 | 87.5 | 90.6 | 88.0 |
| 2nd richest | 38.0 | 35.9 | 45.9 | 41.3 | 86.8 | 83.3 | 93.5 | 89.0 |
| richest | 47.3 | 21.2 | 49.7 | 47.2 | 87.3 | 84.6 | 93.9 | 89.7 |
| Total | 43.3 | 30.6 | 49.1 | 45.4 | 85.3 | 84.0 | 91.4 | 87.8 |
| Institutional delivery* |  |  |  |  |  |  |  |  |
| poorest | 13.0 | 36.8 | 48.7 | 29.8 | 85.7 | 88.3 | 94.4 | 89.2 |
| 2nd poorest | 17.6 | 32.7 | 61.4 | 37.9 | 86.4 | 82.8 | 97.1 | 91.1 |
| middle | 21.0 | 46.8 | 69.7 | 42.7 | 89.3 | 94.5 | 96.9 | 93.1 |
| 2nd richest | 28.1 | 51.5 | 74.1 | 48.1 | 89.4 | 95.0 | 98.9 | 92.8 |
| richest | 52.0 | 60.6 | 92.7 | 69.4 | 95.5 | 93.3 | 99.3 | 96.8 |
| Total | 24.2 | 44.5 | 66.4 | 43.3 | 88.8 | 90.9 | 96.9 | 92.2 |

Note: * only among women reporting any child delivery during the last one year

1. Inflation adjusted using CPI (IW) [↑](#footnote-ref-1)
