# Supplementary material for: Did the poor gain from India’s health policy interventions? Evidence from benefit-incidence analysis, 2004–2018
Source: Int J Equity Health. 2021 Jul 10;20:159. doi: 10.1186/s12939-021-01489-0 (PMC8272306; doi:10.1186/s12939-021-01489-0)
Supplement: Supplementary file 2 — Additional file2: Table S5. Percentage of inpatient and outpatient healthcare shared by the poorest 20% and the richest 20% population groups and concentration indices in 2004 and 2018. Table S6. Percentage of maternity healthcare shared by the poorest 20% and the richest 20% population groups and concentration indices in 2004 and 2018. Table S7. Percentage of healthcare subsidy by the poorest 20% and the richest 20% population groups and concentration indices in 2004 and 2018. Table S8. Percentage of maternity care subsidy received by poorest 20% and richest 20% population and concentration indices in 2004 and 2018. Table S9. Percentage of inpatient and outpatient healthcare shared by the poorest 20% and the richest 20% population groups and concentration indices in 2018. Table S10. Percentage of healthcare subsidy by the poorest 20% and the richest 20% population groups and concentration indices in 2018. Table S11. Distribution of Reasons for not availing healthcare treatment in Government Facilities Across Different Income Classes. [file 12939_2021_1489_MOESM2_ESM.docx]

Supplementary Table S-IV: Percentage of pregnant women utilising ante-natal care, post-natal care and institutional delivery across high focus, high focus north east and other states, 2004 and 2018

|  | 2004 |  |  |  | 2017-18 |  |  |  |
| --- | --- | --- | --- | --- | --- | --- | --- | --- |
|  | High Focus | North East | Other | All India | High Focus | North East | Other | All India |
| Pre natal care |  |  |  |  |  |  |  |  |
| poorest | 55.6 | 70.0 | 81.9 | 67.7 | 95.8 | 97.1 | 96.9 | 96.3 |
| 2nd poorest | 55.1 | 73.5 | 86.9 | 69.6 | 96.1 | 93.5 | 98.5 | 97.1 |
| middle | 60.7 | 54.7 | 85.7 | 71.8 | 98.0 | 91.4 | 97.4 | 97.5 |
| 2ndrichest | 62.3 | 76.3 | 90.8 | 74.6 | 95.9 | 90.1 | 99.5 | 96.9 |
| richest | 74.4 | 86.0 | 90.1 | 81.5 | 96.7 | 98.6 | 99.5 | 97.9 |
| All | 61.0 | 72.5 | 86.9 | 72.6 | 96.4 | 94.2 | 98.1 | 97.0 |
| Post natal care |  |  |  |  |  |  |  |  |
| poorest | 48.9 | 38.2 | 51.5 | 49.7 | 82.5 | 86.8 | 90.1 | 85.6 |
| 2nd poorest | 42.2 | 32.4 | 50.7 | 45.8 | 86.3 | 76.0 | 90.7 | 87.9 |
| middle | 41.1 | 25.3 | 47.7 | 43.6 | 85.6 | 87.5 | 90.6 | 88.0 |
| 2nd richest | 38.0 | 35.9 | 45.9 | 41.3 | 86.8 | 83.3 | 93.5 | 89.0 |
| richest | 47.3 | 21.2 | 49.7 | 47.2 | 87.3 | 84.6 | 93.9 | 89.7 |
| Total | 43.3 | 30.6 | 49.1 | 45.4 | 85.3 | 84.0 | 91.4 | 87.8 |
| Institutional delivery* |  |  |  |  |  |  |  |  |
| poorest | 13.0 | 36.8 | 48.7 | 29.8 | 85.7 | 88.3 | 94.4 | 89.2 |
| 2nd poorest | 17.6 | 32.7 | 61.4 | 37.9 | 86.4 | 82.8 | 97.1 | 91.1 |
| middle | 21.0 | 46.8 | 69.7 | 42.7 | 89.3 | 94.5 | 96.9 | 93.1 |
| 2nd richest | 28.1 | 51.5 | 74.1 | 48.1 | 89.4 | 95.0 | 98.9 | 92.8 |
| richest | 52.0 | 60.6 | 92.7 | 69.4 | 95.5 | 93.3 | 99.3 | 96.8 |
| Total | 24.2 | 44.5 | 66.4 | 43.3 | 88.8 | 90.9 | 96.9 | 92.2 |

Note: * only among women reporting any child delivery during the last one year

Table S-V: Percentage of inpatient and outpatient healthcare shared by the poorest 20% and the richest 20% population groups and concentration indices in 2004 and 2018

| **Quintile Groups** | **2004** |  |  |  | **2018** |  |  |  |
| --- | --- | --- | --- | --- | --- | --- | --- | --- |
|  | **HF States** | **HFNE States** | **Other States** | **All states** | **HF States** | **HFNE States** | **Other States** | **All states** |
| **Inpatient Public** |  |  |  |  |  |  |  |  |
| Poorest 20% | 13.77 | 20.32 | 20.15 | 19.03 | 20.37 | 19.7 | 23.41 | 18.07 |
| Upper middle 20% | 18.3 | 18.9 | 21.25 | 17.24 | 16.59 | 18.15 | 21.95 | 18.9 |
| middle 20% | 19.56 | 19.34 | 21.98 | 20.26 | 19.16 | 19.51 | 22.51 | 20.55 |
| Lower middle 20% | 21.91 | 21.11 | 21.77 | 21.65 | 20.58 | 26.15 | 17.87 | 20.65 |
| Richest 20% | 26.46 | 20.34 | 14.85 | 21.82 | 23.29 | 16.5 | 14.27 | 21.83 |
| *CI* | 0.1417 | 0.0194 | -0.0315 | 0.074 | 0.0967 | 0.1649 | -0.0800 | 0.049 |
| *SE* | 0.0118 | 0.0292 | 0.0100 | 0.007 | 0.0126 | 0.0137 | 0.0095 | 0.007 |
| **Inpatient Private** |  |  |  |  |  |  |  |  |
| Poorest 20% | 10.95 | 17.43 | 12.14 | 10.01 | 15.11 | 13.62 | 14.84 | 11.9 |
| Upper middle 20% | 14.57 | 7.99 | 14.95 | 13.43 | 12.28 | 8.69 | 16.01 | 15.44 |
| middle 20% | 17.37 | 12.93 | 21.23 | 16.57 | 13.95 | 13.1 | 18.48 | 17.5 |
| Lower middle 20% | 21.55 | 22.7 | 22.97 | 22.48 | 23.15 | 20.69 | 22.57 | 22.02 |
| Richest 20% | 35.56 | 38.95 | 28.71 | 37.51 | 35.52 | 43.91 | 28.1 | 33.14 |
| *CI* | 0.2457 | 0.2913 | 0.2240 | 0.283 | 0.2484 | 0.3569 | 0.1708 | 0.245 |
| *SE* | 0.0110 | 0.0489 | 0.0074 | 0.006 | 0.0113 | 0.0241 | 0.0079 | 0.006 |
| **Outpatient Public** |  |  |  |  |  |  |  |  |
| Poorest 20% | 18.49 | 25.64 | 19.78 | 18.47 | 20.42 | 21.6 | 23.02 | 15.95 |
| Upper middle 20% | 15.57 | 24.01 | 21.11 | 16.11 | 18.62 | 27.03 | 19.24 | 19 |
| middle 20% | 16.72 | 16.11 | 19.52 | 18.35 | 16.51 | 19.02 | 21.16 | 17.11 |
| Lower middle 20% | 20.41 | 17.93 | 21.58 | 20.36 | 19.86 | 19.73 | 19.32 | 19.62 |
| Richest 20% | 28.8 | 16.3 | 18.01 | 26.71 | 24.59 | 12.62 | 17.26 | 28.32 |
| *CI* | 0.1587 | -0.1095 | 0.0202 | 0.102 | 0.0919 | -0.1007 | 0.0040 | 0.125 |
| *SE* | 0.0168 | 0.0380 | 0.0129 | 0.01 | 0.0241 | 0.0613 | 0.0137 | 0.011 |
| **Outpatient Private** |  |  |  |  |  |  |  |  |
| Poorest 20% | 13.17 | 23.83 | 12.63 | 14.09 | 15.32 | 17.65 | 13.27 | 11.64 |
| Upper middle 20% | 16.19 | 23.99 | 14.82 | 14.27 | 15.48 | 8.23 | 14.72 | 16.66 |
| middle 20% | 20.78 | 13.22 | 18.54 | 18.53 | 17.58 | 15.82 | 18.4 | 18.5 |
| Lower middle 20% | 22.67 | 18.07 | 24.88 | 21.8 | 22.51 | 23.98 | 22.15 | 21.3 |
| Richest 20% | 27.2 | 20.9 | 29.12 | 31.31 | 29.11 | 34.32 | 31.46 | 31.9 |
| *CI* | 0.1431 | -0.0457 | 0.1963 | 0.191 | 0.1635 | 0.1617 | 0.1794 | 0.229 |
| *SE* | 0.0087 | 0.0296 | 0.0067 | 0.005 | 0.0158 | 0.0775 | 0.0095 | 0.008 |

Table S-VI: Percentage of maternity healthcare shared by the poorest 20% and the richest 20% population groups and concentration indices in 2004 and 2018

| **Quintile Groups** | **2004** |  |  |  | **2018** |  |  |  |
| --- | --- | --- | --- | --- | --- | --- | --- | --- |
|  | **HF States** | **HFNE States** | **Other States** | **All states** | **HF States** | **HFNE States** | **Other States** | **All states** |
| **Pre-natal care in Public Facilities** |  |  |  |  |  |  |  |  |
| Poorest 20% | 22.38 | 23.35 | 28.5 | 24.91 | 28.61 | 25.22 | 29.33 | 26.87 |
| Upper middle 20% | 20.95 | 21.43 | 22.82 | 20.56 | 16.66 | 17.67 | 27.23 | 23.74 |
| middle 20% | 19.48 | 15.67 | 19.88 | 20.45 | 20.32 | 20.52 | 19.78 | 19.38 |
| Lower middle 20% | 22.48 | 18.39 | 19.87 | 17.74 | 20.91 | 26.62 | 15.9 | 17.55 |
| Richest 20% | 14.72 | 21.17 | 8.93 | 16.34 | 13.5 | 9.97 | 7.75 | 12.47 |
| *CI* | *-0.022* | *0.001* | *-0.174* | -0.090 | *-0.126* | *-0.073* | *-0.241* | -0.212 |
| *SE* | *0.021* | *0.051* | *0.017* | 0.012 | *0.013* | *0.027* | *0.014* | 0.008 |
| **Pre-natal care in Private Facilities** |  |  |  |  |  |  |  |  |
| Poorest 20% | 16.85 | 18.86 | 13.34 | 17.11 | 17.14 | 8.53 | 16.79 | 14.31 |
| Upper middle 20% | 17.93 | 14.98 | 17.36 | 16.68 | 12.11 | 7.48 | 17.67 | 16.65 |
| middle 20% | 18.49 | 16.04 | 19.84 | 20.49 | 17.44 | 9.88 | 20.82 | 19.77 |
| Lower middle 20% | 21.73 | 13.6 | 25.28 | 23.04 | 23.19 | 37.94 | 22.11 | 23.91 |
| Richest 20% | 24.99 | 36.52 | 24.18 | 22.67 | 30.11 | 36.17 | 22.61 | 25.36 |
| *CI* | *0.073* | *0.197* | *0.138* | *0.114* | *0.153* | *0.354* | *0.073* | *0.146* |
| *SE* | *0.025* | *0.103* | *0.021* | *0.017* | *0.024* | *0.053* | *0.016* | *0.013* |
| **Institutional delivery in Public Facilities** |  |  |  |  |  |  |  |  |
| Poorest 20% | 17.49 | 18.98 | 26.41 | 22.43 | 29.61 | 23.05 | 29.46 | 27.5 |
| Upper middle 20% | 13.97 | 17.57 | 26.41 | 24.98 | 17.28 | 19.29 | 26.52 | 23.85 |
| middle 20% | 23.71 | 27.71 | 19.67 | 22.82 | 20.15 | 18.39 | 21.74 | 19.45 |
| Lower middle 20% | 23.22 | 21.05 | 19.88 | 16.79 | 20.6 | 29 | 15.64 | 16.94 |
| Richest 20% | 21.61 | 14.69 | 7.63 | 12.98 | 12.36 | 10.27 | 6.64 | 12.26 |
| *CI* | *0.104* | *0.042* | *-0.193* | *-0.034* | *-0.142* | *-0.050* | *-0.258* | *-0.221* |
| *SE* | *0.042* | *0.069* | *0.021* | *0.019* | *0.012* | *0.026* | *0.010* | *0.007* |
| **Institutional delivery in Private Facilities** |  |  |  |  |  |  |  |  |
| Poorest 20% | 11.97 | 17.39 | 13.61 | 14.72 | 17.3 | 12.9 | 16.42 | 15.29 |
| Upper middle 20% | 11.37 | 21.43 | 15.05 | 12.21 | 11.68 | 8.98 | 18.35 | 16.66 |
| middle 20% | 17.6 | 21.02 | 18.52 | 19.01 | 14.24 | 8.45 | 18.21 | 18.64 |
| Lower middle 20% | 21.18 | 7.49 | 27.71 | 26.18 | 23.91 | 20.52 | 23.4 | 22.34 |
| Richest 20% | 37.88 | 32.67 | 25.11 | 27.87 | 32.86 | 49.15 | 23.61 | 27.07 |
| *CI* | *0.286* | *0.068* | *0.183* | *0.261* | *0.176* | *0.370* | *0.092* | *0.164* |
| *SE* | *0.037* | *0.185* | *0.024* | *0.020* | *0.024* | *0.061* | *0.014* | *0.012* |
| **Post-natal care in Public Facilities** |  |  |  |  |  |  |  |  |
| Poorest 20% | 22.04 | 28.83 | 29.65 | 26.31 | 26.82 | 25 | 29.48 | 27.24 |
| Upper middle 20% | 20.71 | 18.43 | 25.93 | 22.41 | 16.01 | 18.46 | 26.93 | 23.91 |
| middle 20% | 19.64 | 17.71 | 18.91 | 22.48 | 18.81 | 18.26 | 21.08 | 19.77 |
| Lower middle 20% | 20.87 | 21.23 | 16.59 | 14.77 | 21.35 | 28 | 16.24 | 17.11 |
| Richest 20% | 16.74 | 13.8 | 8.93 | 14.04 | 17 | 10.27 | 6.27 | 11.96 |
| *CI* | *-0.035* | *-0.072* | *-0.215* | *-0.119* | *-0.142* | *-0.063* | *-0.250* | *-0.218* |
| *SE* | *0.031* | *0.074* | *0.022* | *0.017* | *0.012* | *0.027* | *0.012* | *0.008* |
| **Post-natal care in Private Facilities** |  |  |  |  |  |  |  |  |
| Poorest 20% | 26.46 | 30.12 | 18.84 | 24.91 | 29.21 | 13.24 | 16.6 | 15.79 |
| Upper middle 20% | 21.11 | 19.79 | 16.77 | 17.99 | 17.67 | 12.77 | 18.18 | 17.16 |
| middle 20% | 17 | 15.57 | 18.73 | 19.46 | 19.56 | 9.05 | 18.69 | 18.5 |
| Lower middle 20% | 18.14 | 18.79 | 25.3 | 20.6 | 20.6 | 20.85 | 22.26 | 22.3 |
| Richest 20% | 17.27 | 15.72 | 20.37 | 17.04 | 12.95 | 44.1 | 24.28 | 26.25 |
| *CI* | *-0.069* | *-0.129* | *0.066* | *-0.037* | *0.129* | *0.300* | *0.086* | *0.135* |
| *SE* | *0.025* | *0.126* | *0.028* | *0.020* | *0.022* | *0.068* | *0.014* | *0.012* |

Table S-VII: Percentage of healthcare subsidy by the poorest 20% and the richest 20% population groups and concentration indices in 2004 and 2018

| **Quintile Groups** | **2004** |  |  |  | **2018** |  |  |  |
| --- | --- | --- | --- | --- | --- | --- | --- | --- |
|  | **HF States** | **HFNE States** | **Other States** | **All States** | **HF States** | **HFNE States** | **Other States** | **All states** |
| **Inpatient care** |  |  |  |  |  |  |  |  |
| Poorest 20% | 10.95 | 12.10 | 13.35 | 14.59 | 12.78 | 13.25 | 21.06 | 13.1 |
| Upper middle 20% | 14.15 | 15.25 | 17.95 | 13.83 | 13.73 | 13.90 | 17.26 | 17.52 |
| middle 20% | 16.59 | 23.08 | 21.80 | 20.49 | 18.75 | 15.52 | 19.38 | 21.16 |
| Lower middle 20% | 20.50 | 19.68 | 22.69 | 21.61 | 19.86 | 24.40 | 19.12 | 21.54 |
| Richest 20% | 37.81 | 29.89 | 24.21 | 29.48 | 34.89 | 32.93 | 23.17 | 26.68 |
| Total | 100 | 100 | 100 | 100 | 100 | 100 | 100 | 100 |
| CI | *0.329* | *0.210* | *0.117* | *0.247* | *0.202* | *0.181* | *0.150* | *0.202* |
| SE | *0.016* | *0.042* | *0.013* | *0.010* | *0.013* | *0.029* | *0.011* | *0.009* |
| **Outpatient care** |  |  |  |  |  |  |  |  |
| Poorest 20% | 12.47 | 16.34 | 17.93 | 15.22 | 16.13 | 25.43 | 19.21 | 16.95 |
| Upper middle 20% | 13.56 | 18.57 | 17.38 | 13.29 | 19.61 | 3.60 | 16.83 | 16.59 |
| middle 20% | 13.23 | 13.15 | 16.20 | 16.69 | 12.69 | 36.30 | 21.94 | 18.4 |
| Lower middle 20% | 26.68 | 24.09 | 20.75 | 23.02 | 23.55 | 12.16 | 18.60 | 17.9 |
| Richest 20% | 34.05 | 27.85 | 27.75 | 31.78 | 28.02 | 22.52 | 23.42 | 30.16 |
| Total | 100 | 100 | 100 | 100 | 100 | 100 | 100 | 100 |
| CI | *0.297* | *0.085* | *0.102* | *0.205* | *0.055* | *0.178* | *0.067* | *0.181* |
| SE | *0.029* | *0.052* | *0.018* | *0.014* | *0.022* | *0.099* | *0.013* | *0.018* |

Table S-VIII Percentage of maternity care subsidy received by poorest 20% and richest 20% population and concentration indices in 2004 and 2018

| **Quintile Groups** | **2004** | | | | **2018** | | | |
| --- | --- | --- | --- | --- | --- | --- | --- | --- |
|  | **HF States** | **HFNE States** | **Other States** | **All states** | **HF States** | **HFNE States** | **Other States** | **All states** |
| Prenatal care |  |  |  |  |  |  |  |  |
| Poorest 20% | 16.20 | 1.90 | 20.73 | 19.05 | 26.39 | 11.39 | 25.80 | 20.68 |
| Upper middle 20% | 25.64 | 2.88 | 25.91 | 16.75 | 13.91 | 10.74 | 28.08 | 22.94 |
| middle 20% | 18.49 | 11.66 | 20.10 | 19.78 | 16.12 | 23.87 | 18.68 | 19.18 |
| Lower middle 20% | 22.57 | 47.67 | 20.61 | 20.54 | 27.90 | 34.85 | 17.79 | 19.16 |
| Richest 20% | 17.09 | 35.89 | 12.64 | 23.88 | 15.68 | 19.14 | 9.65 | 18.04 |
| Total | 100 | 100 | 100 | 100 | 100 | 100 | 100 | 100 |
| CI | *0.085* | *0.436* | *0.070* | *0.014* | *0.118* | *0.213* | *0.066* | *0.128* |
| SE | *0.013* | *0.030* | *0.008* | *0.008* | *0.005* | *0.009* | *0.005* | *0.003* |
| Institutional delivery |  |  |  |  |  |  |  |  |
| Poorest 20% | 17.03 | 2.29 | 22.94 | 16.48 | 23.40 | 3.73 | 24.57 | 18.01 |
| Upper middle 20% | 18.65 | 19.47 | 22.12 | 22.69 | 16.07 | 12.49 | 22.90 | 19.74 |
| middle 20% | 19.80 | 1.10 | 18.57 | 20.97 | 18.17 | 19.69 | 19.80 | 21.19 |
| Lower middle 20% | 26.55 | 60.80 | 24.14 | 15.18 | 24.52 | 44.33 | 19.69 | 21.37 |
| Richest 20% | 17.97 | 16.33 | 12.23 | 24.68 | 17.84 | 19.76 | 13.04 | 19.69 |
| Total | 100 | 100 | 100 | 100 | 100 | 100 | 100 | 100 |
| CI | *0.134* | *0.377* | *0.117* | *0.171* | *0.159* | *0.296* | *0.142* | *0.288* |
| SE | *0.010* | *0.027* | *0.009* | *0.007* | *0.008* | *0.011* | *0.008* | *0.005* |
| Postnatal care |  |  |  |  |  |  |  |  |
| Poorest 20% | 20.52 | 2.44 | 27.34 | 19.12 | 24.23 | 29.79 | 27.76 | 23.20 |
| Upper middle 20% | 22.09 | 7.20 | 20.43 | 19.17 | 22.17 | 6.89 | 26.85 | 24.25 |
| middle 20% | 17.19 | 8.08 | 23.31 | 22.50 | 17.99 | 17.19 | 20.25 | 18.96 |
| Lower middle 20% | 22.65 | 61.88 | 20.23 | 16.49 | 20.17 | 23.39 | 17.87 | 18.39 |
| Richest 20% | 17.54 | 20.40 | 8.68 | 22.72 | 15.45 | 22.73 | 7.27 | 15.19 |
| Total | 100 | 100 | 100 | 100 | 100 | 100 | 100 | 100 |
| CI | *0.105* | *0.396* | *0.045* | *0.135* | *0.028* | *0.056* | *0.044* | *0.063* |
| SE | *0.028* | *0.037* | *0.013* | *0.014* | *0.008* | *0.018* | *0.006* | *0.005* |

Table S-IX: Percentage of inpatient and outpatient healthcare shared by the poorest 20% and the richest 20% population groups and concentration indices in 2018

|  | 2004 | | | | | | 2018 | | | | | |
| --- | --- | --- | --- | --- | --- | --- | --- | --- | --- | --- | --- | --- |
| **Quintile Groups** | **Public** | | | **Private** | | | **Public** | | | **Private** | | |
|  | **Communicable diseases** | **Non-communicable diseases** | **All** | **Communicable diseases** | **Non-communicable diseases** | **All** | **Communicable diseases** | **Non-communicable diseases** | **All** | **Communicable diseases** | **Non-communicable diseases** | **All** |
| **Inpatient** | | | | | | | | | | | | |
| Poorest 20% | 24.04 | 17.25 | 19.03 | 12.56 | 9.29 | 10.01 | 20.54 | 16.6 | 18.07 | 11.9 | 11.9 | 11.9 |
| Upper middle 20% | 18.7 | 16.72 | 17.24 | 14.77 | 13.05 | 13.43 | 18.98 | 18.85 | 18.9 | 14.62 | 15.84 | 15.44 |
| middle 20% | 20.76 | 20.08 | 20.26 | 17.77 | 16.22 | 16.57 | 19.7 | 21.05 | 20.55 | 18.95 | 16.8 | 17.5 |
| Lower middle 20% | 19.24 | 22.51 | 21.65 | 22.05 | 22.61 | 22.48 | 20.75 | 20.58 | 20.65 | 23.34 | 21.39 | 22.02 |
| Richest 20% | 17.26 | 23.44 | 21.82 | 32.85 | 38.83 | 37.51 | 20.03 | 22.91 | 21.83 | 31.2 | 34.08 | 33.14 |
| *CI* | -0.148 | 0.113 | 0.074 | 0.122 | 0.301 | 0.283 | -0.136 | 0.074 | 0.049 | 0.118 | 0.246 | 0.245 |
| *SE* | 0.012 | 0.008 | 0.007 | 0.011 | 0.006 | 0.006 | 0.008 | 0.009 | 0.007 | 0.008 | 0.008 | 0.006 |
| **Outpatient** | | | | | | | | | | | | |
| Poorest 20% | 20.99 | 17.31 | 18.47 | 18.49 | 11.77 | 14.09 | 23.08 | 12.2 | 15.95 | 16.91 | 8.34 | 11.64 |
| Upper middle 20% | 18.32 | 15.09 | 16.11 | 17.41 | 12.61 | 14.27 | 21.3 | 17.79 | 19 | 19.59 | 14.82 | 16.66 |
| middle 20% | 21.6 | 16.85 | 18.35 | 20.23 | 17.64 | 18.53 | 16.31 | 17.54 | 17.11 | 21.86 | 16.4 | 18.5 |
| Lower middle 20% | 17.75 | 21.57 | 20.36 | 22.02 | 21.69 | 21.8 | 19.12 | 19.89 | 19.62 | 19.33 | 22.53 | 21.3 |
| Richest 20% | 21.34 | 29.18 | 26.71 | 21.85 | 36.3 | 31.31 | 20.19 | 32.59 | 28.32 | 22.32 | 37.91 | 31.9 |
| *CI* | -0.043 | 0.164 | 0.102 | 0.037 | 0.266 | 0.191 | -0.048 | 0.211 | 0.125 | 0.048 | 0.342 | 0.229 |
| *SE* | 0.017 | 0.011 | 0.010 | 0.005 | 0.006 | 0.005 | 0.018 | 0.013 | 0.011 | 0.009 | 0.010 | 0.008 |

Table S-X: Percentage of healthcare subsidy by the poorest 20% and the richest 20% population groups and concentration indices in 2018

| **Quintile Groups** | **2004** | | | **2018** | | |
| --- | --- | --- | --- | --- | --- | --- |
|  | **Communicable diseases** | **Non-communicable diseases** | **All** | **Communicable diseases** | **Non-communicable diseases** | **All** |
| **Inpatient care** | | | | | | |
| Poorest 20% | 18.39 | 13.85 | 14.59 | 17.74 | 11.94 | 13.10 |
| Upper middle 20% | 14.29 | 13.63 | 13.83 | 15.78 | 17.84 | 17.52 |
| middle 20% | 27.36 | 19.54 | 20.49 | 21.97 | 21.15 | 21.16 |
| Lower middle 20% | 18.49 | 22.09 | 21.61 | 20.47 | 21.80 | 21.54 |
| Richest 20% | 21.47 | 30.88 | 29.48 | 24.04 | 27.27 | 26.68 |
| Total | 100 | 100 | 100 | 100 | 100 | 100 |
| CI | *0.007* | *0.273* | *0.247* | *0.077* | *0.222* | *0.202* |
| SE | *0.022* | *0.010* | *0.010* | *0.017* | *0.017* | *0.009* |
| **Outpatient care** | | | | | | |
| Poorest 20% | 19.34 | 13.82 | 15.22 | 25.51 | 13.82 | 16.95 |
| Upper middle 20% | 15.97 | 12.56 | 13.29 | 17.41 | 15.71 | 16.59 |
| middle 20% | 21.80 | 15.26 | 16.69 | 15.43 | 20.06 | 18.40 |
| Lower middle 20% | 20.00 | 24.24 | 23.02 | 14.77 | 19.09 | 17.90 |
| Richest 20% | 22.89 | 34.12 | 31.78 | 26.88 | 31.32 | 30.16 |
| Total | 100 | 100 | 100 | 100 | 100 | 100 |
| CI | *0.023* | *0.257* | *0.205* | *-0.013* | *0.460* | *0.181* |
| SE | *0.025* | *0.016* | *0.014* | *0.055* | *0.020* | *0.018* |

Table S-XI: Distribution of Reasons for not availing healthcare treatment in Government Facilities Across Different Income Classes

|  | Poorest 20% | Upper middle 20% | Middle 20% | Lower middle 20% | Richest 20% | Total |
| --- | --- | --- | --- | --- | --- | --- |
| Inpatient (2018) |  |  |  |  |  |  |
| Required specific services not available | 17.93 | 16.15 | 14.09 | 17.3 | 12.87 | 15.17 |
| Available but quality not satisfactory/doctor not available | 39.23 | 39.14 | 37.37 | 37.93 | 34.77 | 37.13 |
| Quality satisfactory but facility too far | 6.18 | 4.4 | 4.38 | 5.05 | 5.16 | 5.01 |
| Quality satisfactory but involves long waiting | 9.9 | 14.69 | 15.51 | 12.59 | 15.87 | 14.19 |
| Financial constraint | 0.08 | 0.56 | 0.27 | 0.2 | 0.16 | 0.24 |
| Preference for a trusted doctor/hospital | 19.59 | 19.12 | 23.87 | 22.49 | 25.62 | 22.9 |
| Others | 7.08 | 5.95 | 4.52 | 4.43 | 5.53 | 5.36 |
| Total | 100 | 100 | 100 | 100 | 100 | 100 |
| Outpatient (2018) |  |  |  |  |  |  |
| Required specific services not available | 8.32 | 7.4 | 6.64 | 6.29 | 8.02 | 7.33 |
| Available but quality not satisfactory/doctor not available | 25.59 | 27.22 | 26.34 | 27.11 | 28.49 | 27.25 |
| Quality satisfactory but facility too far | 20.32 | 14.43 | 12.04 | 11.4 | 7.85 | 11.93 |
| Quality satisfactory but involves long waiting | 14.27 | 16.91 | 19.45 | 16.44 | 18.29 | 17.41 |
| Financial constraint | 1.58 | 0.38 | 0.52 | 0.48 | 0.18 | 0.5 |
| Preference for a trusted doctor/hospital | 22.92 | 27.61 | 29.59 | 31.53 | 32.97 | 29.98 |
| Others | 7.01 | 6.04 | 5.43 | 6.75 | 4.2 | 5.61 |
| Total | 100 | 100 | 100 | 100 | 100 | 100 |
